# [O─I─O]^+^ Halogen‐Bonded Complexes of Pyridine *N*‐Oxides

**DOI:** 10.1002/anie.4671643

**Published:** 2026-07-30

**Authors:** Rakesh Puttreddy, Sergi Burguera, Antonio Frontera, Kari Rissanen

**Affiliations:** ^1^ University of Jyvaskyla Jyvaskyla Finland; ^2^ Departament de Química Universitat De Les Illes Balears Palma de Mallorca (Baleares) Spain

**Keywords:** 3‐center‐4‐electron, Barluenga, halogen bond, halogen(I), halonium, iodine(I), *N*‐oxide

## Abstract

Eight [O─I─O]^+^ halogen‐bonded complexes derived from pyridine *N*‐oxides have been characterized by single‐crystal x‐ray diffraction analysis. In these 3‐*center*‐4‐*electron* (3*c*4*e*) halogen‐bonded systems, iodine(I), in its simplest halogen bond (XB) donor form, functions as a bidentate electron acceptor, symmetrically engaging two monodentate *N*‐oxide O‐atoms. The I^+^─O bond lengths of 2.172(17)‐2.227(6) Å represent the shortest XBs reported for *N*‐oxide‐based systems. Solution complexation was studied by ^1^H,^15^N HMBC NMR spectroscopy. With the crystallographic verification of the previously elusive [O─I─O]^+^ motif, Density Functional Theory calculations were performed to compare the electronic and bonding characteristics of [N─I─N]^+^, [O─I─O]^+^, and [S─I─S]^+^ systems. The computations reveal a distinct behaviour for the [O─I─O]^+^ complexes. Unlike nitrogen and sulfur counterparts, the *N*‐oxide O‐atom in [O─I─O]^+^ complexes retain an active electron residual viz. second lone pair, rendering these complexes susceptible to secondary interactions such as hydrogen bonds. This distinctive bonding environment establishes [O─I─O]^+^ complexes as a unique class within 3*c*4*e* halogen‐bonded systems and highlights their potential utility in the rational design of organic reactions and supramolecular materials.

## Graphical abstract

The missing puzzle of 3‐*center*‐4‐*electron* halogen‐bonded complexes viz. [O─I─O]^+^ of pyridine *N*‐oxides are structurally characterized by x‐ray diffraction. Monodentate *N*‐oxide O‐atom acts as the halogen bond acceptor. Their solution complexations are characterized by ^15^N NMR spectroscopy. Computational studies reveal distinct bonding characteristics relative to [N─I─N]^+^ and [S─I─S]^+^ analogues.

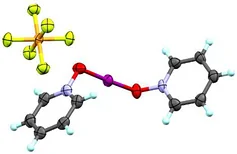

## 1 Introduction

Halogen bonding (XB) [1] is a noncovalent interaction in which a halogen atom (X), acting as an electrophilic center, engages with a nucleophilic site such as a lone pair of electrons. This interaction originates from an anisotropic distribution of electron density around the halogen atom, leading to the formation of a region of positive electrostatic potential, known as the σ‐hole [2]. The magnitude of the σ‐hole is influenced by the polarizability of the halogen and when the halogen atom is covalently attached to electron‐withdrawing groups, such as haloperfluoroarenes and cationic frameworks [3]. Extensive research has also been conducted on a wide range of XB acceptors, including N, S, O, and P atoms [4]. These studies have provided fundamental insights into strength, directionality, and cooperativity, facilitating the establishment of predictive design principles for molecular engineering [5, 6], anion recognition [7], biology [8], and functional materials [9]. Among the various XB acceptors, pyridinic systems in particular have attracted considerable attention due to the N‐atom's well‐defined lone pair of electrons, tunable basicity, and structural versatility. Prominent pyridinic XB motifs include neutral C─X···N [4], N─X···N [10, 11, 12], and cationic 3‐*center*‐4‐*electron* (3*c*4*e*) [N─X─N]^+^ [13] XBs, each offering unique bonding characteristics and applications. For instance, C─I···N XBs, typically formed between iodoperfluoroalkanes/arenes and pyridines, are exploited in functional materials due to their reversible robustness [9]. In contrast, [N─X─N]^+^ motif stabilizes the otherwise highly reactive halogen(I) through a 3*c*4*e* XB. This interaction underpins the formation of halogen(I) or Barluenga's complexes (e.g., [bis(pyridine)iodine(I)]BF_4_), which has found broad utility as halogenating [14], cyclization [15] and oxidizing agents [16]. These cationic XB motifs have also been used in the construction of supramolecular capsules [17] cages [18], and porous halogen‐bonded frameworks [19, 20]. It is worth mentioning that halogen(I) cations cannot exist as a free solid‐state cation due to their extreme instability. Their stabilization requires coordination by two Lewis bases arranged in a linear geometry, typically involving nitrogen and sulphur, to form [S/N─X─N/S]^+^ type complexes, in which the central halogen(I) ion adopts a *p*_x_^2^ *p*_y_^2^ *p*_z_^0^ orbital configuration with the vacant *p*_z_^0^ orbital as a bidentate electron acceptor [13].

The broad applicability of N‐heterocycles in materials design arises from the predictable monodentate coordination bonding or XB behavior of the N‐atom. This characteristic enables reliable synthetic control and facilitates correlations between their chemistries across solid, solution, and gas phases. In contrast, O‐atom‐based XB acceptor systems, particularly those employing pyridine *N*‐oxides (PyNOs), have received considerably less attention. This limited focus is primarily attributed to the three lone pairs of electrons on the *N*‐oxide O‐atom, which permit multidentate coordination bonds [12, 21, 22, 23], XB and hydrogen bond (HB) interactions [24, 25, 26]. Such versatility introduces significant structural complexity and raises questions regarding the comparability of solution and solid‐state architectures.

Our investigations [27, 28, 29, 30, 31], together with literature studies [32, 33, 34, 35, 36, 37, 38, 39, 40], have addressed these questions by examining the denticity of *N*‐oxides, their bonding modes with silver(I) and XB donors, and their coordination and XB strengths relative to pyridinic systems. The *N*‐oxide O‐atom can form strong monodentate N─X···O^−^─N^+^ (X═Br, I) XB modes with N‐haloimides, as the N─X bonds generally remain intact during complexation [27, 28]. These XBs exhibit strengths comparable to those observed in [N─X─N]^+^ systems [27, 28]. Yet, *N*‐oxide O‐atoms frequently break N─X bonds in N‐haloimides, resulting in the formation of N─H···O^−^─N^+^ hydrogen‐bonded complexes [27, 28]. This behavior underscores the inherent difficulty in stabilizing monodentate XB modes even with N‐haloimides. Consequently, even though first cationic [S/N─X─N/S]^+^ systems [41, 42] were structurally characterized nearly five decades ago (Figure 1), isolating single crystals of analogous [O─X─O]^+^ complexes of PyNOs, comprising monodentate *N*‐oxide bonding modes, for x‐ray crystallography has proven challenging.

**FIGURE 1:**
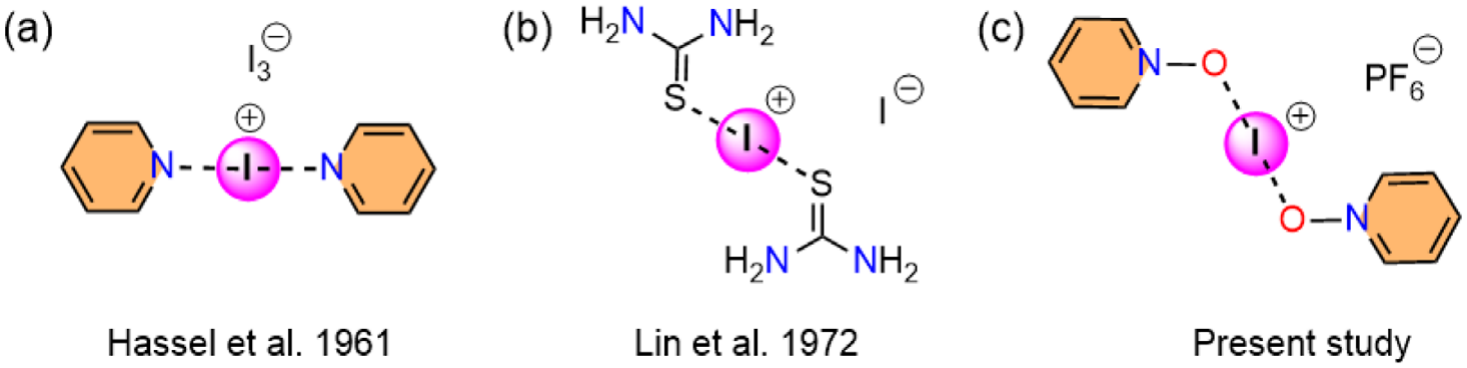
X‐Ray crystal structure examples of cationic 3‐*center*‐4‐*electron* halogen‐bonded systems: (a) [N─I─N]^+^ complexes reported by Hassel et al. in 1961 (CCDC No: 1240761) [41], (b) [S─I─S]^+^ complexes described by Lin et al. in 1972 (CCDC No: 1181004) [42], and (c) [O─I─O]^+^ complexes in the present study.

In this study, we report the synthesis and x‐ray crystal structures of [O─I─O]^+^ halogen‐bonded complexes involving *N*‐oxide O‐atoms as XB acceptors. These complexes are characterized by solution NMR, and select complexes are studied by density functional theory (DFT) computations. In these [O─I─O]^+^ complexes, iodine(I) in its simplest form acts as a bidentate XB donor, while each *N*‐oxide O‐atom functions as a monodentate XB acceptor, forming the shortest I^+^─O XBs of its kind.

## 2 Results and Discussions

Eight [O─I─O]^+^ complexes (**1‐I – 7‐I**) of pyridine *N*‐oxide (**1**), 2‐methylpyridine *N*‐oxide (**2**), 3‐methylpyridine *N*‐oxide (**3**), 4‐methylpyridine *N*‐oxide (**4**), 2‐methoxypyridine *N*‐oxide (**5**), 4‐methoxypyridine *N*‐oxide (**6**) and 3‐bromopyridine *N*‐oxide (**7**) were synthesized and structurally characterized by single‐crystal x‐ray diffraction (Figures 2, 3a, 3b, and S4–S12). Iodine(I) complexes are labelled using the format **n‐I** (*n* = PyNOs = **1**–**7**, e.g., **1‐I**), while the corresponding silver(I) complexes as **n‐Ag** (e.g., **1‐Ag**).

**FIGURE 2:**
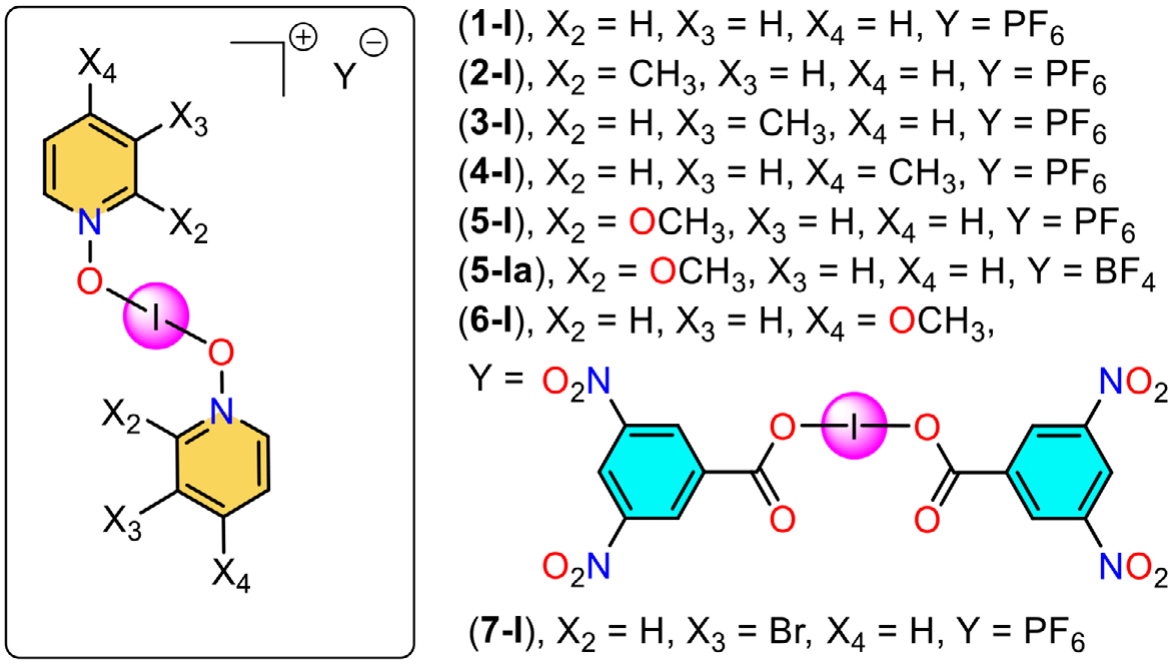
Chemical structures of [O─I─O]^+^ complexes of *N*‐oxides. X_2_ denotes an ortho‐substitution, X_3_ a meta‐substitution, and X_4_ a para‐substitution.

**FIGURE 3:**
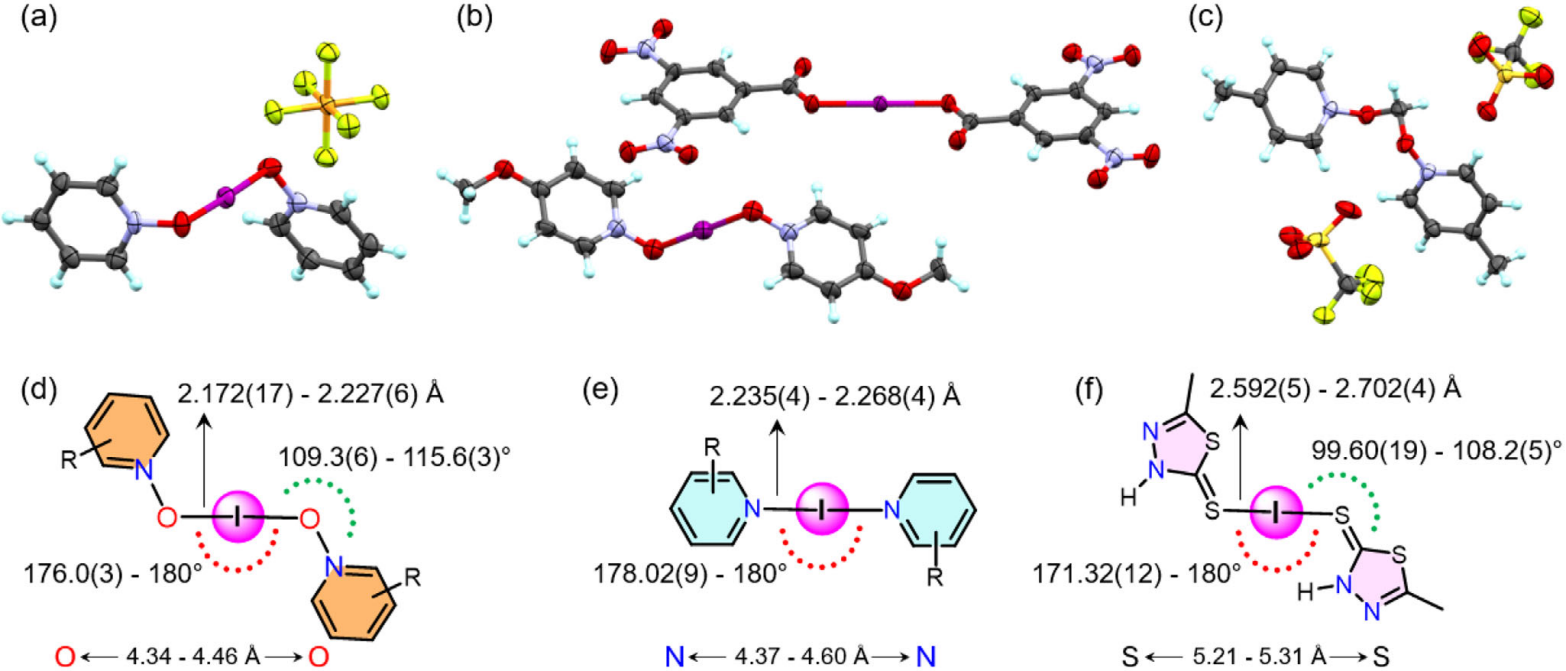
X‐Ray crystal structures of (a) **1‐I** (b) **6‐I** and (c) {(**4**)_2_CH_2_}(OTf)_2_ with the thermal displacement parameter at 50% probability level. Color key: Purple = iodine, orange = phosphorus, yellow = sulfur, lime green = fluorine, red = oxygen, blue = nitrogen, grey = carbon, light cyan = hydrogen. (d–f) Summary of selected XB parameters of 3*c*4*e* halogen‐bonded complexes, (d) [O─I─O]^+^, (e) [N─I─N]^+^ and (f) [S─I─S]^+^.

Typically, the synthesis of [N─I─N]^+^ complexes involves combining 2.0 equivalents of pyridines with 1.0 equivalent of a silver(I) salt to generate the discrete [N─Ag─N]^+^ complex. Subsequent Ag^+^ → I^+^ exchange is achieved by addition of ≈1.0–1.1 equivalents of elemental iodine, which converts [N─Ag─N]^+^ into the corresponding [N─I─N]^+^ and concomitantly produces AgI precipitate. After removal of AgI by filtration, the filtrate is employed directly for crystallization and spectroscopic studies. In these systems, pyridinic‐N acts as a monodentate electron donor, enabling efficient Ag^+^ → I^+^ cation exchange and transformation of [N─Ag─N]^+^ discrete coordination complex into [N─I─N]^+^ discrete halogen‐bonded complex. The discrete nature of [N─Ag─N]^+^ complexes has been extensively confirmed through x‐ray crystallography using a broad range of pyridines and silver(I) salts (see also Figures S13–S18) [43]. In contrast, the formation of [O─I─O]^+^ complexes derived from PyNOs follows the same synthetic framework but differs in structural and mechanistic detail. PyNOs‐silver(I) complexes in aprotic solvents form gels [44], and thus not all silver(I) precursor complexes yielded crystalline material. Only three complexes, viz. **1‐Ag**, **2‐Ag** and **4‐Ag** afforded single crystals suitable for x‐ray crystallography, and all three were identified as polymeric complexes (see Figures S23–S25). These observations suggest that the Ag^+^ → I^+^ exchange most likely proceeds through a polymeric silver(I) complex. The coordination numbers of silver(I) across these three polymers span four to eight, and the PyNOs display denticities of one to three (see Figure S26). This variability is consistent with the d^10^ configuration of silver(I) and its classification as a soft Lewis acid according to Hard and Soft Acids and Bases (HSAB) theory [45]. As such, silver(I) forms relatively weak interactions with hard donors such as *N*‐oxide O‐atoms, favouring polymeric, rather than discrete silver(I) complexes. Notably, crystallization attempts of the **1‐Ag** – **7‐Ag** PyNO complexes from acetonitrile resulted in four silver(I) structures. They all consistently demonstrated polymeric structures in the solid state (see Figures S27–S30). Within these structures, the silver(I) centers exhibit coordination numbers ranging from four to six, and there are no bis‐coordinate Ag(I)‐PyNOs complexes (see Figures S31 and S32). These experimental observations rule out the linear two‐coordinate silver(I) complexes predicted by the DFT‐optimized model [46]. No such [O─Ag─O]^+^ species have been found to exist, nor have they been isolated or crystallographically characterized. Thus, the proposed mechanism for the formation of the [O─I─O]^+^ species detected in solution [46] by NMR is invalid. It is also worth mentioning that, e.g., a 2:1 equivalent ratio of PyNO:AgPF_6_ undergoes Ag^+^ → I^+^ exchange with ≈0.43‐0.65 equivalents of elemental iodine to afford desired [O─I─O]^+^ complexes (for more details, see Supporting Information). All these observations suggest that the [O─Ag─O]^+^ → [O─I─O]^+^ transformation cannot be rationalized as straightforwardly [46] as the well‐established [N─Ag─N]^+^ → [N─X─N]^+^ pathway [13].

Single crystals of **1‐I, 2‐I, 3‐I, 4‐I**, and **6‐I** were synthesized using Schlenk techniques, rigorously dried solvents, and storage at −24°C. Even under these conditions, crystallization remains challenging, with only a limited number of crystals forming over several weeks (for more details, see Supporting Information). In contrast, **5‐I, 5‐Ia,** and **7‐I** were synthesized under ambient conditions using HPLC‐grade solvents. Notably, **5‐I** affords crystals overnight at −4°C in either dichloromethane or dichloroethane. In **5‐I** and **5‐Ia**, the *N*‐oxide exhibits positional disorder, and efforts to obtain disorder‐free crystal structures were unsuccessful. Nevertheless, among the eight structurally characterized complexes, six contain PF_6_^−^ as the counterion, one contains BF_4_^−^, and in one case the counterion itself is a carboxylate that participates in formation of an anionic [O─I─O]^−^ XB motif. Several PyNOs and silver(I) salts were explored to broaden the scope of [O─I─O]^+^ complexes and to evaluate substituent effects on XBs. But the high reactivity of the [O─I─O]^+^ motif usually led to isolation of hydrogen‐bonded species of the type such as [PyNO‐H]^+^Y^−^ and [(PyNO)_2_‐H]^+^Y^−^ (Y = counter anion), a mixture of [PyNO‐H]^+^Y^−^/[(PyNO)_2_‐H]^+^Y^−^ and PyNO‐I_2,_ or uncomplexed PyNOs (see Figures S34–S53). In addition, crystals of an unusual product arising from alkylation of the *N*‐oxide by dichloromethane have been characterized (Figure 3c). The N─O–alkylation chemistry is known in organic synthesis [47, 48].

The I^+^─O bond distances observed for the [O─I─O]^+^ span a narrow range of 2.172(17)–2.227(6) Å, only ≈0.04‐0.08 Å shorter than the I^+^─N distances typically observed in [N─I─N]^+^ complexes formed from the corresponding parent pyridines (see Table S1) [43]. In contrast, the I^+^─S distances within [S─I─S]^+^ complexes [49, 50, 51, 52, 53, 54, 55, 56, 57, 58] are substantially longer, by ≈0.42–0.48 Å, reflecting the intrinsically larger van der Waals radii of S‐atom and its correspondingly weaker donor properties compared to O‐atom. Establishing meaningful correlations between I^+^─O bond lengths and substituent effects is challenging, as the observed variations are minimal (see Table 1). This limited variability can be attributed to the strong attractive interaction between the PyNOs O‐atom lone pairs and the iodine(I) *p*_z_ orbital. Such behavior is a defining characteristic of 3*c*4*e* halogen‐bonded systems [13]. The O─I─O bond angles (176.0(3)‐180°) closely mirror those of [N─I─N]^+^ (178.02(9)‐180°) and [S─I─S]^+^ (171.32(12)‐180°) complexes, as the linearity is dictated by the arrangement of the iodine(I) *p*_z_ orbital along the O···I^+^···O axis, consistent with the general symmetry rules governing the 3*c*4*e* bonding. Because N‐ and O‐atoms possess similar atomic sizes, the O···O distances within [O─I─O]^+^ (4.34–4.46 Å) are comparable to those observed in [N─I─N]^+^ complexes (4.37–4.60 Å). However, larger S···S separations in [S─I─S]^+^ systems (5.21–5.31 Å) naturally follow from the increased size of S‐atom. Nevertheless, these structural similarities indicate that replacement of S/N‐atom with O‐atom preserves the fundamental geometric requirements of strong 3*c*4*e* halogen bonding.

**TABLE 1:** Selected x‐ray crystallography bond parameters and ^1^H,^15^N HMBC NMR‐based absolute Δδ^15^N_coord_ values of [O─I─O]^+^ halogen‐bonded complexes.

Code	O−I (Å)	I−O (Å)	O···O (Å)	∠O−I−O (°)	|Δδ^15^N_coord_|^a^
	∠*N−O−I (°)*	∠*I−O−N (°)*			(ppm)
**1‐I**	2.206(5)	2.202(5)	4.41	177.8(2)	30
	*109.6(3)*	*111.5(4)*			
**2‐I**	2.199(4)	2.199(4)	4.40	180	28
	*111.4(3)*	*111.4(3)*			
**3‐I**	2.192(4)	2.212(4)	4.40	178.74(17)	34
	*113.6(3)*	*115.6(3)*			
**4‐I**	2.199(7)	2.194(7)	4.39	176.0(3)	27
	*113.5(5)*	*113.3(5)*			
**5‐I** ^b^	2.172(17)	2.172(17)	4.34	180	29
	*111.8(12)*	*111.8(12)*			
**5‐Ia** ^b^	2.227(6)	2.227(6)	4.46	180	28
	*111.7(5)*	*111.7(5)*			
**6‐I**	2.192(8)	2.192(8)	4.38	180	29^c^
	*113.8(6)*	*113.8(6)*			
**7‐I**	2.206(2)	2.206(2)	4.41	180	30
	*114.23(17)*	*114.23(17)*			

^a^ Δδ^15^N_coord_ = Δδ^15^N_complex_ − Δδ^15^N_ligand_.

^b^ The *N*‐oxide ligands are disordered in these complexes.

^c^ The *N*‐oxide nitrogen of **6‐I** did not exhibit a ^15^N signal. As a result, iodine(I) complex of 4‐methoxypyridine *N*‐oxide was prepared with PF_6_^−^ as the counteranion, and the reported ^15^N NMR data corresponds to this species.

A notable structural distinction, however, is that unlike the classical [N─I─N]^+^ complexes, well‐known for their nearly perfect coplanarity, the [O─I─O]^+^ systems lack a coplanar arrangement. In [N─I─N]^+^ complexes, the coplanarity enables efficient overlap between the pyridinic‐N lone pairs and iodine(I) σhole, thereby enhancing πdelocalization across the (aromatic)N···I···N(aromatic) framework and stabilizing the XBs. The deviation from coplanarity in [O─I─O]^+^ complexes may therefore influence crystal packing efficiency and intermolecular interactions. The N─O─I bond angles in [O─I─O]^+^ complexes range from 109.6(3)° to 115.6(3)°, a bent geometry that closely parallels that observed in S‐based [S─I─S]^+^ complexes (99.60(19)‐108.2(5)°). This similarity underscores that while electron donor‐acceptor characteristics (I^+^─O = soft‐hard vs. I^+^─S = soft‐soft) modulate bond metrics, the overall angular distortions remain consistent with established structural expectations for iodine(I) systems.

Packing analysis of **1‐I – 7‐I** revealed that PyNOs O‐atoms consistently exhibit multidentate coordination behavior, functioning either in bidentate (one I^+^─O^−^─N^+^ XB and one C─H···O^−^─N^+^ HB) or tridentate (one I^+^─O^−^─N^+^ XB, two C─H or C···O^−^─N^+^ HBs) interaction modes (see Figures S54 and S55). Such ancillary HBs significantly enhance stabilizations of solid‐state structures and reflect a well‐documented intrinsic polydentate character of PyNOs [24, 25, 26]. This feature is crucial when rationalizing the thermodynamic and structural robustness of [O─I─O]^+^ complexes and should not be overlooked when comparing them to N‐ or S‐based XB systems. Among the eight structures, **6‐I** is particularly noteworthy (Figure 3b). In this structure, the O‐atom of the carboxylate does not engage in the formation of a cationic [O─I─O]^+^ motif but instead exclusively generates an anionic [O─I─O]^−^ species counterbalanced by the [O─I─O]^+^ XB motif of *N*‐oxide ligand. The I^+^─O distances of 2.203(6) Å of the anionic framework closely align with literature carboxylatebased [O─I─O]^−^ systems (2.164(6)–2.203(2) Å) [59, 60, 61] and alkoxide‐based [O─I─O]^−^ system (2.185(7) Å) [62] and are ≈0.1 Å longer than the I^+^─O bond observed in the *N*‐oxide‐based [O─I─O]^+^ system. The ─NO_2_ groups of [O─I─O]^−^ framework in **6‐I** exhibit a noteworthy structural feature. The N‐atom engages in four formal bonds, viz. a single bond to one O‐atom bearing a −1 charge, a double bond to the second O‐atom, and a σ‐bond to the aromatic ring. Resonance renders the two N─O bonds equivalent and delocalizes the negative charge over both O‐atoms, while the nitrogen retains a +1 formal charge. This electronic arrangement makes ─NO_2_ one of the strongest electron‐withdrawing substituents and potentially an electron acceptor in the presence of a suitable electron donor. In the packing structure of **6‐I**, the electron belt of the iodine(I) is sandwiched between two ─NO_2_ group N‐atoms at distances of 3.62 Å (see Figure S55). Additionally, N···O contacts (2.90 Å) are observed between a ─NO_2_ group associated with the carboxylate and the methoxy O‐atom of 4‐methoxypyridine *N*‐oxide. Although these contacts arise from packing forces, their persistent solid‐state structural presence highlights two important aspects: (i) the ─NO_2_ group nitrogen can serve as an electron acceptor, and (ii) the iodine(I) electron belt can function as an electron donor.

The ^15^N NMR coordination shift, defined as the difference between the 𝛿^15^N chemical shift of a halogen‐bonded complex and its corresponding free PyNO/ligand (Δδ^15^N_coord_ = Δδ^15^N_complex_ − Δδ^15^N_ligand_), was employed to probe solution XB complexation. Larger absolute coordination shifts (|Δδ^15^N_coord_|) reflect greater perturbation of the nitrogen electron density upon complex formation and have previously proven to be a reliable spectroscopic descriptor for elucidating structural features in well‐defined [N─I─N]^+^ systems [13, 43]. All NMR samples were prepared under Schlenk conditions using dried CD_2_Cl_2_. It is noteworthy that numerous crystallization experiments were required to obtain eight crystal structures, underscoring the inherent complexity of *N*‐oxide chemistry. Consequently, solution NMR should be regarded as complementary to crystal structures. Despite these challenges, some patterns emerged from the ^15^N NMR data. For PyNO‐silver(I) complexes, |Δδ^15^N_coord_| values range from 1 to 9 ppm. However, these values should be interpreted with caution, as PyNOs‐silver(I) systems form gels or precipitation in CD_2_Cl_2_, rendering the corresponding ^15^N NMR data unreliable. In contrast, the [O─I─O]^+^ complexes exhibit larger |Δδ^15^N_coord_| values, spanning 27–34 ppm. Importantly, the |Δδ^15^N_coord_| values of protonated (PyNOH)^+^PF_6_^−^ differ from [O─I─O]^+^ complexes (see Table S4), thereby excluding protonation effects and confirming the formation of genuine halogen‐bonded species in solution. Further insight arises from comparing solid‐state and solution data. Complex **1‐I**, when prepared under Schlenk conditions in dichloroethane, crystallizes in the triclinic space group *P*‐*1*, whereas the same species prepared directly in the NMR tube in CD_2_Cl_2_ crystallizes in monoclinic space group *P*2_1_/n. Both crystal forms incorporate solvent molecules in their asymmetric units (see Figures S4 vs. S5). Furthermore, **5‐I** crystals dissolved in CD_2_Cl_2_ display a 𝛿^15^N value of −135 ppm and a |Δδ^15^N_coord_| of 29 ppm, identical to the values obtained when **5‐I** was generated in situ in the NMR tube (Figure 4). Note that the in‐situ NMR tube preparation of **5‐I** also produced single crystals of the corresponding [O─I─O]^+^ complex. However, the structure is severely disordered and was therefore excluded from the present study. Nevertheless, these observations collectively demonstrate that the 𝛿^15^N chemical shifts observed for **1‐I** and **5‐I** could reflect the discrete [O─I─O]^+^ halogen‐bonded complexes present in solution. Although the |Δδ^15^N_coord_| values for the [O─I─O]^+^ complexes are smaller than [N─I─N]^+^ system (e.g., ca. 110 ppm for [(pyridine)_2_I]^+^ in CD_2_Cl_2_ versus ca. 30 ppm for **1‐I** in CD_2_Cl_2_; Table S4), this difference can be attributed to their distinct binding modes. In [N─I─N]^+^ complexes, the pyridinic‐N participate directly in XB, leading to significant perturbations of the ^15^N chemical environment. In contrast, in [O─I─O]^+^ complexes, the N‐atom is not the primary XB acceptor, resulting in comparatively smaller |Δδ^15^N_coord_| values. Nevertheless, the shifts observed for the [O─I─O]^+^ systems exceed those reported for the strongest known N─I···O^−^─N^+^ halogen bonds formed between N‐iodoimides and PyNOs [27, 28]. This trend is consistent with the high electrophilicity of iodine(I), which is widely recognized as one of the strongest XB donors [13, 43]. Accordingly, the comparatively large |Δδ^15^N_coord_| values measured for the [O─I─O]^+^ motif provide convincing NMR evidence for the presence of 3*c*4*e* XBs in solution.

**FIGURE 4:**
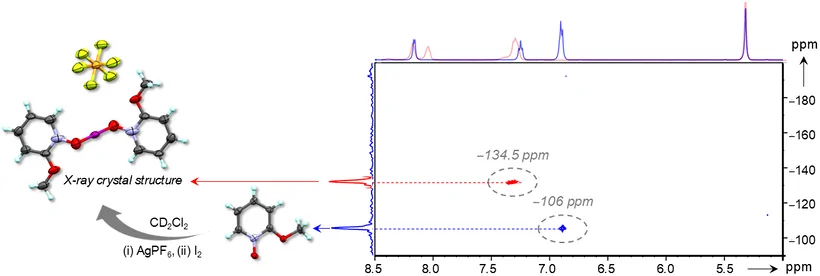
^1^H, ^15^N HMBC NMR spectra of **5‐I**. (right‐side) The blue trace corresponds to the uncomplexed *N*‐oxide and the red trace corresponds to **5‐I**. (left‐side) X‐Ray crystal structure of **5‐I** with the thermal displacement parameter at 50% probability level. Color key: Purple = iodine, orange = phosphorus, yellow = sulfur, lime green = fluorine, red = oxygen, blue = nitrogen, grey = carbon, light cyan = hydrogen. Notes (i) the coordinates for uncomplexed **5** were obtained from iodine(I) complex. (ii) The red contour of **5‐I** is made using Microsoft PowerPoint.

The successful x‐ray crystallography of the previously elusive [O─I─O]^+^ complex permits a comparative analysis using molecular electrostatic potential mapping, the Quantum Theory of Atoms in Molecules (QTAIM), and the Extended Transition State Natural Orbitals for Chemical Valence (ETS‑NOCV) [63] decomposition (Figures 5, 6, and S89–S91). These methods were applied to structurally characterized halogen‐bonded systems, viz. [O─I─O]^+^ of PyNO, [N─I─N]^+^ of pyridine, and of [S─I─S]_1_^+^ of 2‐mercapto‐5‐methyl‐1,3,4‐thiadiazole (Figure 5c) [56]. In their crystal structures, [O─I─O]^+^ and [N─I─N]^+^ cationic XB motifs are counterbalanced by PF_6_ anions, whereas the [S─I─S]_1_^+^ is with the FeCl_4_ anion. Any specific anion‑mediated perturbations to the XBs were not investigated in the present study. To further examine the influence of the aromatic backbone on the [S─I─S]_1_^+^ motif, additional calculations were performed on a related [S─I─S]_1_^+^ motif derived from 3‐methylbenzothiazole‐2‐thione [50]. The aromatic framework did not produce significant changes in the key electronic descriptors. Therefore, the corresponding computational data are provided in the Supporting Information. However, the presence of a second S‐atom within a thiadiazole framework provides additional stabilization to the [S─I─S]_1_^+^ motif through secondary ^+^I─S interactions, a feature that is absent in [N─I─N]^+^ and [O─I─O]^+^ systems. Therefore, to provide a more meaningful comparison with these secondary interactions‐free systems, the [S─I─S]_2_^+^ motif was investigated using a simple [H_2_C═S─I─S═CH_2_]^+^ halogen‐bonded model that excludes such interactions. For clarity, the notation [S─I─S]^+^ is used as a general descriptor, whereas [S─I─S]_1_^+^ refers specifically to the motif derived from thiadiazole crystal structure, and [S─I─S]_2_^+^ corresponds to computed [H_2_C═S─I─S═CH_2_]^+^ XB complex.

**FIGURE 5:**
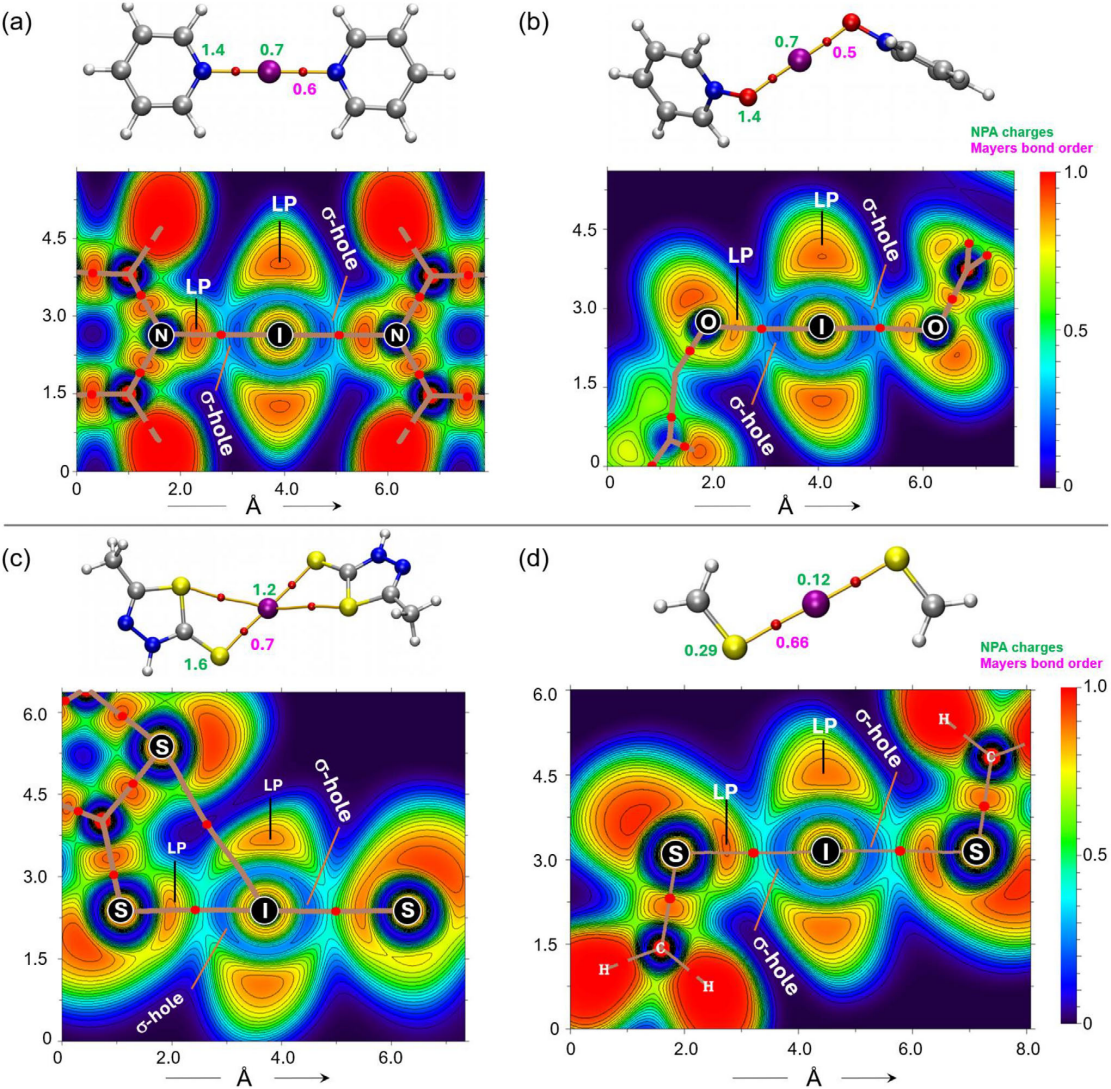
The optimized molecular geometries and corresponding 2DELF map of (a) [N─I─N]^+^ (b) [O─I─O]^+^, (c) [S─I─S]_1_^+^, and (d) [S─I─S]_2_^+^ halogen‐bonded complexes. ELF values are color‐coded from low (blue) to high (red) localization. Distinct maxima correspond to lone‐pair (LP) basins on the donor atoms and σhole regions on the iodine(I), illustrating the spatial distribution of electron localization and the donor‐acceptor nature of the halogen bonding interaction. NPA charges are indicated in green font, and Mayer bond orders are indicated in magenta font. Level of theory is RI‐PBE0 [66]‐D4 [67]/def2‐TZVP [68, 69] for all analyses.

**FIGURE 6:**
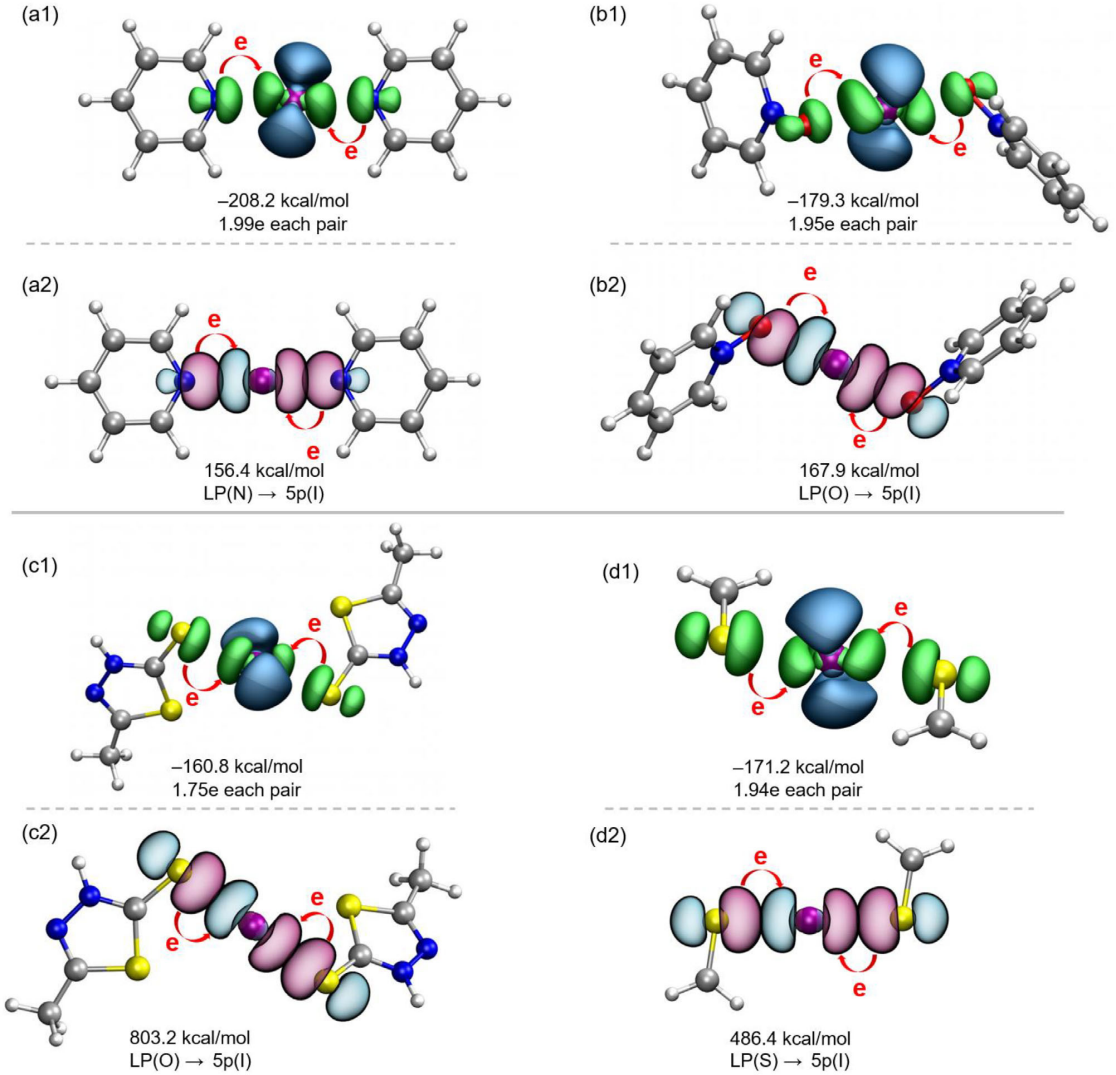
ETSNOCV deformation density plots and associated orbital interaction energies for (a1) [N─I─N]^+^ (b1) [O─I─O]^+^, (c1) [S─I─S]_1_^+^ and (d1) [S─I─S]_2_^+^ halogen‐bonded complexes at the RI‐PBE0‐D4/def2‐TZVP level of theory. Values in parentheses are computed at the ZORA‐PBE0‐D4/ZORA‐def2‐TZVP [72, 73] level of theory. The dominant donor‐acceptor orbital pairs responsible for XB formation are shown using red arrows NBO representations, and second‐order perturbation stabilization energies (E^(2)^) associated with LP → 5p(I) charge‐transfer interactions in (a2) [N─I─N]^+^ (b2) [O─I─O]^+^, (c2) [S─I─S]_1_^+^ and (d2) [S─I─S]_2_^+^ halogen‐bonded complexes. The red arrows indicate the direction of electron donation from the N/O/S‐atom lone pairs to the iodine(I) 5*p* orbitals.

QTAIM analysis focused on bond critical point (BCP) descriptors for the I^+^─O/N/S XBs, namely, the electron density (ρ_BCP_), the Laplacian of the electron density (∇^2^ρ_BCP_), and the kinetic‑to‑potential energy density ratio (−G/V). Together, these parameters quantify the interplay of electrostatic, closed‑shell, and covalent contributions to the XB. In particular, the −G/V ratio is used to classify the nature of chemical bonding, with −G/V > 1 characteristic of closed‐shell (ionic, van der Waals, or dipole‐dipole) interactions, −G/V < 0.5 indicating shared‐shell (covalent) interactions, while 0.5 < −G/V < 1 values correspond to partially covalent interactions. The ETS‑NOCV analyses further deconvolute the donor → acceptor charge‑transfer properties and associated orbital interaction compositions and corresponding energies. Note that ETS‐NOCV focuses on the orbital term, further decomposing it into chemically meaningful contributions [64]. This approach, based on pairs of bonding and antibonding orbitals, enables the extraction of a diatomic‐like picture of chemical bonding.

The MEP analysis revealed that the *p*_x_ *p*_y_‐orbital electron density (known as electron belt) around the iodine(I) follows the order: [S─I─S]_2_^+^ (−78 kcal/mol) > [N─I─N]^+^ (−61 kcal/mol) > [S─I─S]_1_^+^ (−50 kcal/mol) ≈ [O─I─O]^+^ (−51 kcal/mol) (Figure S89). A more visual chemical bonding picture of XBs is provided by Electron Localization Function (ELF) topological studies. All four halogen‐bonded systems exhibit a well‐defined σ‐hole along the *p*_z_ axis, the lateral *p*_x_ *p*_y_ electron‐belt surrounding the iodine(I), and the N/O/S lone pair electron density being directed into the σ‑hole. A key distinguishing feature of the [O─I─O]^+^ and [S─I─S]^+^ motifs is the persistence of substantial electron localization corresponding to the lone pair on the O‐/S‐atoms, even after the monodentate XB formation. This feature is absent in [N─I─N]^+^. This residual electron density renders [O─I─O]^+^ and [S─I─S]^+^ complexes more susceptible to engaging in secondary interactions, leading to a greater propensity for supramolecular reactivity (e.g., Figure 3c). These characteristics likely contribute to the increased difficulty in crystallizing [O─I─O]^+^ and [S─I─S]^+^ complexes compared to the traditional [N─I─N]^+^ complexes. Note that across Mulliken, Löwdin, and Hirshfeld analyses of [N─I─N]^+^, [O─I─O]^+^, and [S─I─S]_1_^+^, the *N*‐oxide O‐atom consistently carries a more negative charge than the N‐ and S‐atoms (see Table S5). For all four halogen‐bonded systems, the −G/V values fall within a narrow range of 0.63–0.70, suggesting I^+^─O/N/S interactions as electrostatic XBs with a significant degree of covalent character. More importantly, the similarity of −G/V ratios across different 3*c*4*e* halogen‐bonded systems involving N‐, O‐, and S‐atoms underscores the robustness and transferable nature of these cationic iodine(I) complexes. Natural population analysis (NPA) charges and Mayer bond orders further corroborate this interpretation (Figure 5a–d). The NPA charges confirm significant charge transfer from the donor atoms to the iodine(I) centre. Mayer bond orders [65] are consistently greater than 0.5, supporting the single‐bond character and dominant covalent nature of the ^+^I─O,^+^I─N, and ^+^I─S interactions.

The energies, charge transfer, and orbital composition from ETS‐NOCV analysis [63] of the three halogen‐bonded systems are given in Table 2. The ETS‐NOCV analysis revealed a clear trend in the orbital interaction energies. The strongest interaction is observed for [N─I─N]^+^ (−208.2 kcal/mol) followed by [O─I─O]^+^ (−179.3 kcal/mol), [S─I─S]_2_^+^ (−171.2 kcal/mol) and [S─I─S]_1_^+^ (−160.8 kcal/mol). Charge transfer follows the similar trend. The N‐atom exhibits the largest charge flow (1.99e), closely followed by O‐atom (1.95e), while a noticeably smaller charge transfer is observed for S‐atoms (1.94e) in [S─I─S]_2_^+^ compared to [S─I─S]_1_^+^ (1.75e). The donor orbitals are dominated by *p*‐character (80.6–89.4%), suggesting the primary role of the *p*‐lone pairs of N/O/S‐atoms in XB formation. The *s*‐character contribution remains minimal (≈1%–5%), while *d*‐orbital participation is negligible (<0.5%) even for the [S─I─S]^+^ system. A systematic reduction in orbital interaction energy and charge transfer from N to S‐atoms suggests decreased donor ability and increased spatial diffuseness of the valence orbitals, and this could be one of the reasons for decreased V_s, min_ on iodine(I) electron‐belt on [O─I─O]^+^ and [S─I─S]_1_^+^ than in [N─I─N]^+^ (Figure S89). Importantly, the ETS‐NOCV results evidently demonstrate that 3*c*4*e* XBs are directional and orbital‐controlled interactions rather than a purely electrostatic phenomenon that is typical of, e.g., C─I···N interactions formed by haloalkanes and N‐heterocycles [70].

**TABLE 2:** Selected energies, charge transfer and orbital composition values obtained from Extended Transition State‐Natural Orbitals for Chemical Valence (ETS–NOCV) decomposition analysis.

XB motif^a^	Energy (kcal/mol)	Charge transfer	Orbital pair	%*s*	%*p*	%*d*
[N─I─N]^+^ ^b^	−208.2	1.99e	I/N	1.1/19.8	98.8/80.6	0.1/0.2
[O─I─O]^+^ ^c^	−179.3	1.95e	I/O	1.0/10.4	98.9/89.4	0.1/0.2
[S─I─S]_1_^+^ ^d^	−160.8	1.75e	I/S	4.9/16.8	94.7/82.9	0.4/0.3
[S─I─S]_2_^+^	−171.2	1.94e	I/S	0.3/11.4	99.4/88.5	0.3/0.1

^a^ X‐Ray crystal structure coordinates are used for the ETS‐NOCV study.

^b^ CCDC number: 1030916 [71].

^c^ CCDC number: 2529433.

^d^ CCDC number: 813538 [56].

Energy decomposition analysis (EDA) of the four XB motifs reveals closely comparable total interaction energies (E_int_) across the series. The [S─I─S]_1_^+^ (−139.6 kcal/mol) incorporating secondary interactions is marginally more stabilized than [N─I─N]^+^ (−130.8 kcal/mol) and [O─I─O]^+^ (−128.5 kcal/mol), while the [S─I─S]_2_^+^ (−118.3 kcal/mol) simple model shows reduced stabilization (Table 3). In all cases, iodine(I) interacts with lone pairs on N/O/S‐atoms, resulting in substantial LP → σ* charge transfer and polarization, as reflected in large orbital interactions (E_o__r__b_) and significant electrostatic (E_ele_) contributions. Only a modest dynamic electron correlation remains after strong I─N/O/S bonds are established, resulting in a relatively small correlation (E_cor_). Similarly, dispersion plays a secondary role because the short I─N/O/S bonds are governed by charge concentration, rendering E_disp_ small but non‐negligible. Notably, secondary ^+^I─S interactions enhance stabilization in the [S─I─S]_1_^+^ motif of thiadiazole, whereas their absence in the model system highlights the intrinsic bonding characteristics of the primary XB motif. This also accounts for the larger orbital‐energy gap between the ETS‐NOCV ΔE_orb_ (Table 2) and the EDA E_orb_ (Table 3) observed for [S─I─S]_1_^+^, relative to [N─I─N]^+^ and [O─I─O]^+^ and [S─I─S]_2_^+^: the EDA E_orb_ term implicitly sums all orbital interactions within the fragment pair, whereas ETS‐NOCV isolates only the single dominant LP → σ* donor‐acceptor pair. For [S─I─S]_1_^+^, the additional ≈35 kcal/mol captured by EDA but not by ETS‐NOCV largely reflects the secondary ^+^I─S orbital interactions inherent to the thiadiazole framework, which are absent in [N─I─N]^+^ and [O─I─O]^+^.

**TABLE 3:** Energy decomposition analysis (EDA, kcal/mol) for 3c4e XB motifs showing total interaction energies (*E*_int_) and individual contributions from exchange–repulsion (*E*_exc‐rep_), electrostatics (*E*_ele_), orbital interactions (*E*_orb_), correlation (*E*_cor_), and dispersion (*E*_disp_). All energies correspond to a single I─N/O/S bond.

XB motif	*E*_int_	*E*_exc‐rep_	*E*_ele_	*E*_orb_	*E*_cor_	*E*_disp_
[N─I─N]^+^	−130.8	178.0	−90.2	−203.9	−12.9	−1.7
[O─I─O]^+^	−128.5	145.7	−80.9	−180.7	−10.9	−1.7
[S─I─S]_1_^+^	−139.6	139.5	−67.8	−195.9	−13.4	−2.0
[S─I─S]_2_^+^	−118.3	137.5	−61.0	−180.9	−12.6	−1.3

Natural Bond Orbital (NBO) analysis was used to provide an orbital‐resolved description of the donor‐acceptor interactions in these iodine(I) systems. The analysis focused on the donation from the N, O, or S lone pairs into the low‐lying 5p acceptor orbital of iodine(I). This approach follows ETS‐NOCV results that identified orbital‐controlled interactions as the primary stabilizing contribution (Figure 6a1–d2). For the [N─I─N]^+^ system, the LP(N) to 5p(I) interaction provides a second‐order stabilization energy, E^(2)^, of 156 kcal/mol, indicating efficient orbital overlap and charge donation from the nitrogen lone pair. In the [O─I─O]^+^system, the LP(O) to 5p(I) interaction increases to 168 kcal/mol (169 kcal/mol using ZORA). This enhancement is attributed to the strong donor capability of the *N*‐oxide O‐atom and improved energetic alignment with the iodine(I) acceptor orbital. NBO visualizations show more pronounced electron density delocalization along the O─I─O axis (Figure 6a2 vs. b2), suggesting increased polarization and a stronger covalent contribution compared to the nitrogen system. In the [S─I─S]_1_^+^ system, a LP(S) to 5p(I) interaction is present, but the small energy gap between the donor and acceptor orbitals (0.81 eV) invalidates the perturbative model. The resulting E^(2)^ value of 803 kcal/mol is unphysically large, confirming that second‐order perturbation theory is not applicable here. For this system, ETS‐NOCV is a more suitable method for analyzing orbital charge transfer. The near‐degeneracy of the donor and acceptor orbitals supports a predominantly covalent description of the [S─I─S]_1_^+^ system. A similar trend is observed for [S─I─S]_2_^+^ motif, with ETS‑NOCV capturing a reasonable charge redistribution with stabilization of −171.2 kcal/mol and an unrealistically large NBO E^(2)^ value of 486.4 kcal/mol due to the small donor‐acceptor energy gap (1.06 eV).

## 3 Conclusion

The [O─I─O]^+^ halogen‐bonded complexes of pyridine *N*‐oxides (PyNOs) have been structurally characterized by single‐crystal x‐ray diffraction following synthesis via an Ag^+^ → I^+^ exchange strategy. While this approach successfully afforded [O─I─O]^+^ complexes, many reactions yielded hydrogen‐bonded species of the type [PyNO‐H]^+^Y^−^ and [(PyNO)_2_‐H]^+^Y^−^ (Y = counter anion). This outcome can be attributed to the *N*‐oxide O‐atom possessing three lone pairs, enabling multidentate coordination. As a result, the synthesis and crystallization of [O─I─O]^+^ complexes are more challenging than well‐established [N─I─N]^+^ complexes. Nevertheless, the I^+^─O bond lengths of 2.172(17)–2.227(6) Å represent the shortest XBs reported for *N*‐oxide‐based systems. X‐Ray crystal structural observations suggested that the Ag^+^ → I^+^ exchange proceeds through polymeric silver(I) precursor complexes. This polymeric‐to‐discrete structural transformation is reasonable considering the d^10^ electronic configuration of Ag^+^, which favors flexible coordination geometries, combined with the multidentate *N*‐oxide O‐atom's bonding propensity. Electron Localization Function (ELF) topological analyses indeed reveal the presence of an unused lone pair on the *N*‐oxide O‐atom in these [O─I─O]^+^ complexes. This residual electron donor capability likely contributes to their tendency toward secondary coordination such as hydrogen bonds, offering a plausible explanation for the limited crystallizability observed for [O─I─O]^+^ systems. Nevertheless, considering that this is the first solid‐state study of [O─I─O]^+^ complexes, it is not possible to draw concrete conclusions on several aspects, including crystallizability and stability with respect to, e.g., *N*‐oxides and solvents. Yet these findings underscore the delicate balance between halogen bonding, silver(I) coordination chemistry, and competing supramolecular interactions that operate to yield desired [O─I─O]^+^ complexes. Future work will focus on expanding the scope using different *N*‐oxides to systematically investigate substituent effects on I^+^─O halogen bond distances, complex stability, as well as isolating and structurally characterizing PyNOs‐silver(I) complexes. The latter task would clarify whether *N*‐oxides can form 2:1 ligand‐to‐metal complexes analogous to those observed for parent pyridines. Furthermore, exploration of solvent effects and reactivity patterns is expected to provide further insight into the mechanisms and stabilization of [O─I─O]^+^ systems.

## 4 Experimental Section

General crystallization procedure: Single crystals for x‑ray crystallography were prepared by initially combining pyridine *N*‐oxide and the corresponding silver(I) salt in a 2:1 molar ratio in either dichloromethane or dichloroethane. To this solution, 0.43–0.65 equivalents of finely ground elemental iodine were added. Two general methodologies were employed for sample preparation: (i) reactions conducted under inert‐atmosphere conditions using Schlenk techniques, and (ii) reactions carried out under ambient conditions. For crystallizations carried out under Schlenk conditions, the reaction mixture was stirred using a magnetic stir bar under an inert atmosphere. The resulting silver(I) iodide precipitate was not removed, and the magnetic stir bar remained in the Schlenk flask. Upon completion of the reaction, the mixture was stored at −24 °C for crystal growth. Crystals suitable for x‑ray diffraction were selected at −25°C–30°C under a stream of nitrogen atmosphere. For crystallizations conducted under ambient conditions, the reaction mixtures were stirred using a benchtop vortex mixer. The silver(I) iodide precipitate formed during the reaction was removed by filtration using a glass pipette fitted with a cotton plug. The resulting solutions were subsequently stored at −4°C to afford single crystals. More experimental details are given in the Supporting Information.

## Author Contributions

**Rakesh Puttreddy**: conceptualization, investigation, methodology, validation, data curation, supervision, writing – review and editing, visualization, writing – original draft, formal analysis. **Sergi Burguera**: software, data curation. **Antonio Frontera**: methodology, validation, visualization, software, formal analysis, data curation, resources, funding acquisition, writing – review and editing. **Kari Rissanen**: funding acquisition, resources, writing – review and editing.

## Conflicts of Interest

The authors declare no conflicts of interest.

## Supporting information



The authors have cited additional references within the Supporting Information [74, 75, 76, 77, 78, 79, 80, 81, 82, 83, 84, 85, 86, 87, 88, 89]. Details of the synthesis, x‐ray diffraction analysis, NMR and Density Functional Theory studies are provided in the Supporting Information. Deposition Numbers 2529433 [for **1‐I**], 2529426 [for **1‐I_1**], 2529442 [for **2‐I**], 2529449 [for **3‐I**], 2529430 [for **4‐I**], 2529416 [for **5‐I**], 2529457 [for **5‐Ia**], 2529443 [for **6‐I**], 2529458 [for **7‐I**], 2529420 [for **{(4)_2_CH_2_}(OTf)_2_**], 2529424 [for {**(3MePy)_2_Ag}PF_6_**], 2529453 [for {**(3MePy)_2_Ag}PF_6__1**], 2529436 [for **{(2MeOPy)_2_Ag}PF_6_**], 2529413 [for **{(2MeOPy)_2_Ag}PF_6__1**], 2529431 [for **{(4MeOPy)_2_Ag}PF_6_**], 2529435 [for **{(3BrPy)_2_Ag}PF_6_**], 2529455 [for **{(3MePy)_2_I}PF_6_**], 2531719 [for **{(2MeOPy)_2_I}PF_6_**], 2529432 [for **{(4MeOPy)_2_I}PF_6_**], 2529440 [for **{(3BrPy)_2_I}PF_6_**], 2529418 [for **1‐Ag**], 2529423 [for **2‐Ag**], 2529439 [for **3‐Ag**], 2529419 [for **4‐Ag**], 2529438 [for **5‐Ag**], 2529428 [for **1‐Ag_1**], 2529437 [for **4‐Ag_1**], 2529446 [for **(TMNOH)CH_3_SO_3_**], 2529444 [for **{(2)_2_H}SbF_6_**], 2529417 [for **{(2)_2_H}PF_6_**], 2529452 [for **(2H)PF_6_**], 2529425 [for {**(3H)3}ClO_4_**], 2529415 [for **(246TriMePyNOH)CH_3_SO_3_**], 2529427 [for {**(246TriMePyNO)_2_H}PF_6_**], 2529451 [for {**(3MeOPyNO)_2_H}PF_6_**], 2529447 [for {**(6)_2_H}PF_6_**], 2529441 [for {**(2Me4MeOPyNO)_2_H}I_3_**], 2529414 [for {**(2Me4MeOPyNO)_2_H}PF_6_**], 2529434 [for {**(3PhPyNO)_2_H**}**PF_6_**], 2529429 [for {**(4EtPyNO)_2_H}PF_6_**], 2529421 [for **(4)4CF_3_BzH**], 2529454 [for {**(7)DNBH**], 2529448 [for {**(3IPyNO)_2_H}PF_6_**], 2529445 [for **IsQNO‐H‐ClO_4_**], 2529422 [for **4DMAPO**], 2529450 [for **1‐I_2_**], 2529456 [for **3‐I_2_**] contains the supplementary crystallographic data for this paper. These data are provided free of charge by the joint Cambridge Crystallographic Data Centre and Fachinformationszentrum Karlsruhe http://www.ccdc.cam.ac.uk/structures Access Structures service.**Supporting File**: anie73916‐sup‐0001‐SuppMat.docx.

## Data Availability

The data that supports the findings of this study are available in the Supporting Information of this article.
