# Supplementary material for: [O─I─O]+ Halogen‐Bonded Complexes of Pyridine N‐Oxides
Source: Angew Chem Int Ed Engl. 2026 Jul 30;65(40):e4671643. doi: 10.1002/anie.4671643 (PMC13618274; doi:10.1002/anie.4671643)
Supplement: Supplementary file 1 [file ANIE-65-e4671643-s001.docx]

Supporting Information for

**[O−I−O]^+^ Halogen-Bonded Complexes of Pyridine *N*-oxides**

**The Supplementary Information file includes:**

Materials and Methods

Supplementary Fig. 1 to 91

Supplementary Table 1 to 5

xyz coordinates of DFT structures

References

1. Genenral information on materials

Pyridine *N*-oxide (95%), 2-methylpyridine *N*-oxide (>96%), 3-methylpyridine *N*-oxide (98%), 4-methylpyridine *N*-oxide (98%), 4-methoxypyridine *N*-oxide (97%), isoquinoline *N*-oxide (98%), 2-methoxypyridine (98%), 4-methoxypyridine (97%), and HPF_6_ (≈50 wt.% in H_2_O) were purchased from Sigma-Aldrich. Pyridine (>99.5%) was purchased from VWR international GmbH. 3-Methylpyridine (>98%), Silver(I) hexafluorophosphate (>98%), silver(I) tetrafluoroborate (98%), and elemental iodine (>99%) were obtained from TCI Chemicals. HPLC-grade dichloromethane and 1,2-dichloroethane purchased from fischer chemicals and dried according to literature procedures.^[1]^ 3,5-Dinitrosilver(I) benzoate was synthesized following a reported method.^[2]^ 2-Methoxypyridine *N*-oxide, 3-methoxypyridine *N*-oxide, 3-phenylpyridine *N*-oxide, 3-bromopyridine *N*-oxide, 3-iodopyridine *N*-oxide, and 4-ethylpyridine *N*-oxide were prepared according to literature procedures.^[3]^

**2. Synthesis of [O–I–O]⁺ halogen-bonded complexes**

**2.1 Synthesis of 1-I**

Pyridine *N*-oxide (194 mg, 2.04 mmol, 2.0 eq) was predried in a lyophilizer overnight, transferred to a Schlenk flask, and further dried under vacuum for 1 hour. Subsequently, dichloroethane (10 mL) was added to the flask, and the mixture was stirred for 15 minutes. An overnight lyophilized AgPF_6_ (245 mg, 0.969 mmol, 0.95 eq) was then introduced, and the suspension was stirred for an additional 20 minutes. Finely ground elemental iodine (111 mg, 0.439 mmol, 0.43 eq) was added, and the mixture was stirred for 5 minutes. The resulting AgI precipitate was not removed, and the magnetic stir bar remained in the flask. The reaction mixture was stored in a refrigerator at −24 °C for two weeks, yielding colorless crystals. In the initial attempt, opening the Schlenk flask at room temperature resulted in rapid decomposition of the crystals. The crystallization was repeated under identical conditions, and the crystals were transferred to the diffractometer under a nitrogen atmosphere and mounted at −30 °C for X-ray diffraction analysis. Despite these precautions, the crystals decomposed immediately upon handling, as observed under the microscope.

**2.2 Synthesis of 2-I**

A mixture of 2-methylpyridine *N*-oxide (5.7 mg, 0.0522 mmol, 2.0 equiv) and silver(I) hexafluorophosphate (6.6 mg, 0.0261 mmol, 1.0 equiv) was taken in HPLC-grade dichloromethane (1.5 mL) and stirred for 1 min using a vortex mixer. Finely ground elemental iodine (3.65 mg, 0.0144 mmol, 0.55 equiv) was then added to the white suspension, and stirring was continued until complete dissolution of iodine was observed. The resulting AgI precipitate was removed by filtration through a cotton plug inserted into a glass pipette. Hexane was diffused into the light-pink filtrate, and the solution was stored at −4 °C. After four weeks, a small number of colourless crystals suitable for single-crystal X-ray diffraction were obtained. The crystal was transferred to the X-ray diffractometer as quickly as possible under room temperature conditions. Prior to this successful attempt, multiple crystallization trials were conducted under varying conditions. These included conventional mixing at room temperature and reactions performed in Schlenk flasks using both HPLC-grade and rigorously dried solvents (acetone, acetonitrile, dichloromethane, dichloroethane, 1,2-dichlorobenzene; anti-solvents tried: hexane, pentane, diethylether). In all cases, the stoichiometric ratio of *N*-oxide to silver(I) salt was maintained at 2:1, while the amount of iodine was varied between 0.25 and 0.85 equiv. None of these trials yielded crystals suitable for X-ray analysis.

**2.3 Synthesis of 3-I**

3-Methylpyridine *N*-oxide (85 mg, 0.779 mmol, 2.0 eq) was predried in a lyophilizer overnight, transferred to a Schlenk flask, and further dried under vacuum for 1 hour. Subsequently, dichloroethane (7 mL) was added to the flask, and the mixture was stirred for 5 minutes. An overnight lyophilized AgPF_6_ (84 mg, 0.331 mmol, 0.85 eq) was then added, and the mixture was stirred for an additional 15 minutes. Finely ground elemental iodine (47 mg, 0.187 mmol, 0.48 eq) was added, and the mixture was stirred for 5 minutes. The AgI precipitate was not removed, and the magnetic stir bar remained in the flask. The reaction mixture was stored in a refrigerator at −24 °C for three weeks, yielding just one colourless crystal on the Schlenk tube wall. Note that prior to this ‘’successful’’ attempt, multiple crystallization trials were conducted under varying conditions. These included conventional mixing at room temperature and reactions performed in Schlenk flasks using both HPLC-grade and rigorously dried solvents (acetone, acetonitrile, dichloromethane, dichloroethane, 1,2-dichlorobenzene; anti-solvents tried: hexane, pentane, diethylether). In all cases, the stoichiometric ratio of *N*-oxide to silver(I) salt was maintained at 2:1, while the amount of iodine was varied between 0.25 and 0.80 equiv. None of these trials yielded crystals suitable for X-ray analysis.

**2.4 Synthesis of 4-I**

4-Methylpyridine N-oxide (137 mg, 1.26 mmol, 2.0 equiv) was predried in a lyophilizer for 6 hours, transferred to a Schlenk flask, and further dried under vacuum for 1 hour. Subsequently, 1,2-dichloroethane (10 mL) was added, and the mixture was stirred for 15 minutes. Lyophilized AgPF₆ (155.5 mg, 0.615 mmol, 0.98 equiv) was then introduced, and the suspension was stirred for an additional 15 minutes. Finely ground elemental iodine (103.6 mg, 0.408 mmol, 0.65 equiv) was added, and stirring continued until complete dissolution of iodine. The AgI precipitate was not removed, and the magnetic stir bar remained in the flask. The reaction mixture was stored at −24 °C for four weeks; the solution retained a light-yellow color and did not yield crystals. The AgI precipitate was subsequently removed by filtration through a cotton plug inserted into a glass pipette. Pentane was then diffused into the light-yellow filtrate, and the solution was stored at −4 °C. After three days, colorless needles/rods and yellow rods were obtained. The colorless crystals correspond to hydrogen-bonded species, whereas the yellow rods represent the targeted halogen-bonded complex (Figure S1). Prior to this successful attempt, multiple crystallization trials were conducted under varying conditions, including conventional mixing at room temperature and reactions performed in Schlenk flasks using both HPLC-grade and rigorously dried solvents (acetone, acetonitrile, dichloromethane, 1,2-dichlorobenzene). Anti-solvents tested included hexane, pentane, and diethyl ether. In all cases, the stoichiometric ratio of *N*-oxide to silver(I) salt was maintained at 2:1, while the amount of iodine was varied between 0.25 and 0.80 equiv. None of these trials yielded crystals suitable for X-ray diffraction analysis.

Supplementary Fig. 1 Microscopic photographs of the complex, **4-I**. The crystals start decompose in ≈15 min.

**2.5 Syntheses of 5-I**

A mixture of 2-methoxypyridine *N*-oxide (6.3 mg, 0.0531 mmol, 2.0 equiv) and AgPF₆ (6.2 mg, 0.0244 mmol, 0.97 equiv) was taken in HPLC-grade 1,2-dichloroethane (1.5 mL) and stirred for 1 min using a vortex mixer. Finely ground elemental iodine (3.8 mg, 0.0151 mmol, 0.60 equiv) was then added, and stirring was continued until complete dissolution of iodine was observed. The resulting AgI precipitate was removed by filtration through a cotton plug inserted into a glass pipette. Pentane was subsequently diffused into the light-yellow filtrate, and the solution was stored at −4 °C. After overnight crystallization, crystals suitable for single-crystal X-ray diffraction were obtained (Figure S2). Using the same procedure with dichloromethane as the solvent, crystals of comparable quality were obtained within overnight.

Crystals obtained from both dichloroethane and dichloromethane exhibited significant disorder and remained stable under ambient conditions. We hypothesize that the observed disorder facilitates an efficient packing, contributing to the stability of these structures. Or simply, stability may arise from multiple factors, including the presence of the *N*-oxide ligand or the specific iodine(I) complex formed with the PF_6_ anion.

Several crystallization trials were conducted to obtain an disorder structure. These included conventional mixing at room temperature and reactions performed in Schlenk flasks using both HPLC-grade and rigorously dried solvents (acetone, acetonitrile, 1,2-dichlorobenzene). Various anti-solvents (hexane, pentane, diethyl ether) and silver(I) salts (AgOTf, AgClO_4_, AgTFA) were also tested. In all cases, the stoichiometric ratio of *N*-oxide to silver(I) salt was maintained at 2:1, while the amount of iodine was varied between 0.35 and 0.80 equiv. Attempts using 0.40 and 0.50 equiv of iodine in dichloromethane and dichloroethane yielded crystals of similar disordered quality, whereas other conditions did not produce any crystalline material.

Supplementary Fig. 2 (a and b) Photographs of the crystal sample of **5-I.** Scale bar not available as the pictures were taken using iphone.

**2.6 Synthesis of 5-Ia**

The reaction mixture was processed following the same protocol as described for the PF_6_ anion analogue using 1,2-dichloroethane as the solvent. A mixture of 2-methoxypyridine *N*-oxide (6.0 mg, 0.0559 mmol, 2.0 equiv), AgBF₄ (5.3 mg, 0.0271 mmol, 0.97 equiv), and finely ground elemental iodine (3.9 mg, 0.0154 mmol, 0.55 equiv) was combined and stirred for 1 minute using a vortex mixer. the resulting AgI precipitate was removed by filtration through a cotton plug inserted into a glass pipette, and pentane was diffused into the filtrate. The solution was stored at −4 °C, and crystals suitable for single-crystal X-ray diffraction were obtained in three days. The structure has a disordered *N*-oxide, similar to that observed in the **5-I**

**2.7 Synthesis of 6-I**

A mixture of 4-methoxypyridine *N*-oxide (9 mg, 0.0719 mmol, 2.0 equiv) and 3,5-dinitrosilver(I) benzoate (11.5 mg, 0.0360 mmol, 1.0 equiv) was taken in HPLC-grade dichloromethane (1.5 mL) and stirred for 1 min using a vortex mixer. Finely ground elemental iodine (5.5 mg, 0.0216 mmol, 0.60 equiv) was then added to the suspension, and stirring was continued until complete dissolution of iodine was observed. The resulting AgI precipitate was removed by filtration through a cotton plug inserted into a glass pipette. Pentane was subsequently diffused into the light-yellow filtrate, and the solution was stored at −4 °C. After two weeks, a small number of crystals suitable for single-crystal X-ray diffraction were obtained. These crystals decompose when taken out of the solvent. However, they were transferred to the X-ray diffractometer as quickly as possible under room temperature conditions. Prior to this successful attempt, multiple crystallization trials were conducted under varying conditions. These included conventional mixing at room temperature and reactions performed in Schlenk flasks using both HPLC-grade and rigorously dried solvents (acetone, acetonitrile, dichloromethane, dichloroethane, 1,2-dichlorobenzene; anti-solvents tried: hexane, pentane, diethylether). In all cases, the stoichiometric ratio of *N*-oxide to silver(I) salt was maintained at 2:1, while the amount of iodine was varied between 0.35 and 0.80 equiv. None of these trials yielded crystals suitable for X-ray analysis.

**2.8 Synthesis of 7-I**

A mixture of 3-bromopyridine *N*-oxide (10 mg, 0.0578 mmol, 2.0 equiv) and silver hexafluorophosphate (7.3 mg, 0.0363 mmol, 1.0 equiv) was taken in HPLC-grade dichloromethane (1.5 mL) and stirred using a vortex mixer for 1 minute. Subsequently, finely ground elemental iodine (4.4 mg, 0.0172 mmol, 0.60 equiv) was added to the suspension, and stirring was continued until complete dissolution of iodine was achieved. The resulting AgI precipitate was removed by filtration through a cotton plug inserted into a glass pipette. The clear, light-pink filtrate was subjected to pentane vapor diffusion, and the solution was stored at −4 °C. After 5 days, a small number of light-yellow crystals suitable for single-crystal X-ray diffraction analysis were obtained. The isolated crystals remained stable under ambient conditions for at least 24 h (Figure S3). The desired complex was successfully obtained in a single crystallization attempt.

Supplementary Fig. 3 (a and b) Microscopic photographs of the **7-I**

**3. General procedure of Pyridine *N*-oxide silver(I) complexes**

A 2:1 molar ratio of *N*-oxide to silver(I) salt on a 10 mg scale with respect to *N*-oxide were taken in schlenk tube and vacuum dried for 1 hour and to this added 5 mL of dichloromethane or dichloroethane at room temperature. The solutions were stirred for 10-15 minutes at room temperature and flasks were left at -4 °C to yield colourless crystals suitable for X-ray diffraction analysis.

**4. X-ray crystallography**

Single crystal X-ray diffraction analysis were carried out using an XtaLAB Synergy R four-circle diffractometer equipped with a Hybrid Pixel Array Detector (detector type: HyPix-Arc 100; Diffraction source type is PhotonJet R (Cu, λ = 1.54184 Å) X-ray source) and Rigaku SuperNova four-circle diffractometer equipped with a Hybrid Pixel Array Detector (detector type: HyPix-Arc 100) and Cu-K_α_ (λ = 1.54184 Å) radiation. CrysAlisPro was used for the data collection and reduction, and the intensities were absorption corrected using a gaussian face index absorption correction method. All structures were solved by intrinsic phasing (SHELXT)^[4]^ and refined by full-matrix least squares on *F*^2^ using the OLEX2,^[5]^ utilizing the SHELXL-2015 module.^[6,7]^ The X-ray single crystal data and CCDC numbers of all new structures are included below. These data can be obtained free of charge via <http://www.ccdc.cam.ac.uk/conts/retrieving.html> (or from the CCDC, 12 Union Road, Cambridge CB2 1EZ, UK; Fax: +44 1223 336033; E-mail: [deposit@ccdc.cam.ac.uk](mailto:deposit@ccdc.cam.ac.uk)).

**4.1 Halogen-bonded complexes of pyridine *N*-oxides**

Supplementary Fig. 4 Crystal structure of **1-I** with the thermal displacement parameter at 50% probability level. Colour Key: purple = iodine, green = chlorine, light orange = phosphorous, light green = fluorine, red = oxygen, blue = nitrogen, dark grey = carbon, and light cyan = hydrogen. Crystallization solvent = dichloroethane.

**Crystal data for 1-I:** CCDC-2529433, C_12_H_14_Cl_2_F_6_IN_2_O_2_P, M = 561.02 gmol^‑1^, colourless plate, 0.14 × 0.08 × 0.03 mm^3^, triclinic, space group *P-1*, a = 8.4771(4) Å, b = 9.4614(5) Å, c = 12.6620(6) Å, α= 69.552(4)°, β = 84.683(4)°, γ = 82.632(4)°, V = 942.46(8) Å^3^, Z = 2, D_calc_ = 1.977 gcm^‑3^, F(000) = 544, µ = 17.420 mm^‑1^, T = 120(1) K, θ_max_ = 76.692°, 13393 total reflections, 11340 with I_o_ > 2σ(I_o_), R_int_ = twin, 13393 data, 239 parameters, 0 restraints, GooF = 1.038, R = 0.0568 and wR = 0.1474 [I_o_ > 2σ(I_o_)], R = 0.0676 and wR = 0.1524 (all reflections), 1.712<d∆ρ < -1.439 eÅ^‑3^.

**Comments on Checkcif file**: There are no A- and B-alerts

Supplementary Fig. 5 (a) Crystal structure of **1-I_1** with the thermal displacement parameter at 50% probability level. Colour Key: purple = iodine, green = chlorine, light orange = phosphorous, light green = fluorine, red = oxygen, blue = nitrogen, dark grey = carbon, and light cyan = hydrogen. Disordered parts were omitted for viewing clarity. Crystallization solvent = CD_2_Cl_2_. (b-d) Pictures of crystals inside the NMR tube (obtained after two weeks from ^15^N NMR measurement) and on the glass slide.

**Crystal data for 1-I_1:** CCDC-2529426, C_11_H_12_Cl_2_F_6_IN_2_O_2_P, M = 547.00 gmol^‑1^, colourless block, 0.2× 0.06× 0.03 mm^3^, monoclinic, space group *P2_1_/n*, a = 7.9644(2)Å, b = 9.3331(2)Å, c = 24.5224(7)Å, α= 90°, β = 91.843(2)°, γ = 90°, V = 821.87(8)Å^3^, Z = 4, D_calc_ = 1.994gcm^‑3^, F(000) = 1056, µ = 18.003mm^‑1^, T = 120(1) K, θ_max_ = 77.658°, 26653 total reflections, 3277 with I_o_ > 2σ(I_o_), R_int_ = 0.0892, 3852 data, 236 parameters, 0 restraints, GooF = 1.311, R = 0.1369 and wR = 0.3566 [I_o_ > 2σ(I_o_)], R = 0.1452 and wR = 0.3592 (all reflections), 5.819<d∆ρ < -1.264eÅ^‑3^.

**Comments on Checkcif file**: There are no A-Alerts. There is a level B-alert. The level B-alert and our response are given below.

PLAT084_ALERT_3_B High wR2 Value (i.e. > 0.25) ................... 0.36 Report

**Author Response: This is due to twin data and unresolved absorption artifacts**

PLAT094_ALERT_2_B Ratio of Maximum / Minimum Residual Density .... 4.60 Report

**Author Response: This is due to absorption artifacts**

PLAT097_ALERT_2_B Large Reported Max. (Positive) Residual Density	5.82 eA-3

**Author Response: This is due to absorption artifacts**

PLAT342_ALERT_3_B Low Bond Precision on C-C Bonds ............... 0.03 Ang.

**Author Response: This is due to twin data and unresolved absorption artifacts**

Supplementary Fig. 6 Crystal structure of **2-I** with the thermal displacement parameter at 50% probability level. Colour Key: purple = iodine, light orange = phosphorous, light green = fluorine, red = oxygen, blue = nitrogen, dark grey = carbon, and light cyan = hydrogen. Crystallization solvent = dichloromethane. Diffusion solvent = hexane.

**Crystal data for 2-I:** CCDC-2529442, C_12_H_14_F_6_IN_2_O_2_P, M = 490.12 gmol^‑1^, colourless block, 0.132 × 0.061 × 0.049 mm^3^, triclinic, space group *P-1*, a = 6.3875(3) Å, b = 6.5958(2) Å, c = 10.2661(4) Å, α= 96.416(3)°, β = 91.312(4)°, γ = 95.247(3)°, V = 427.76(3) Å^3^, Z = 1, D_calc_ = 1.903 gcm^‑3^, F(000) = 238, µ = 16.273 mm^‑1^, T = 120(1) K, θ_max_ = 66.735°, 4713 total reflections, 1505 with I_o_ > 2σ(I_o_), R_int_ = 0.0405, 1511 data, 113 parameters, 0 restraints, GooF = 1.177, R = 0.0367 and wR = 0.1066 [I_o_ > 2σ(I_o_)], R = 0.0372 and wR = 0.1067 (all reflections), 1.802<d∆ρ < -0.817 eÅ^‑3^.

**Comments on Checkcif file**: There are no A- and B-alerts

Supplementary Fig. 7 Crystal structure of **3-I** with the thermal displacement parameter at 50% probability level. Colour Key: purple = iodine, light orange = phosphorous, light green = fluorine, red = oxygen, blue = nitrogen, dark grey = carbon, and light cyan = hydrogen. Crystallization solvent = dichloroethane.

**Crystal data for 3-I:** CCDC- 2529449, C_12_H_14_F_6_IN_2_O_2_P, M = 490.12 gmol^‑1^, colourless block, 0.06 × 0.04 × 0.03mm^3^, monoclinic, space group *C2/c*, a = 23.9230(5) Å, b = 10.8745(3) Å, c = 12.9515(2) Å, α= 90°, β = 99.359(2)°, γ = 90°, V = 3324.49(13) Å^3^, Z = 8, D_calc_ = 1.958 gcm^‑3^, F(000) = 1904, µ = 16.751 mm^‑1^, T = 120(1) K, θ_max_ = 66.484°, 14913 total reflections, 12988 with I_o_ > 2σ(I_o_), R_int_ = twin, 14913 data, 220 parameters, 0 restraints, GooF = 1.036, R = 0.0415 and wR = 0.1114 [I_o_ > 2σ(I_o_)], R = 0.0489 and wR = 0.1152 (all reflections), 1.187<d∆ρ < -0.660 eÅ^‑3^.

**Comments on Checkcif file**: There are no A- and B-alerts

Supplementary Fig. 8 Crystal structure of **4-I** with the thermal displacement parameter at 50% probability level. Colour Key: purple = iodine, light orange = phosphorous, light green = fluorine, red = oxygen, blue = nitrogen, dark grey = carbon, and light cyan = hydrogen. Crystallization solvent = dichloroethane.

**Crystal data for 4-I:** CCDC-2529430, C_12_H_14_F_6_IN_2_O_2_P, M = 490.12 gmol^‑1^, yellow rod/block, 0.19× 0.09× 0.06 mm^3^, monoclinic, space group *P2_1_/c*, a = 11.6405(3)Å, b = 17.9149(4)Å, c = 8.2064(2)Å, α= 90°, β = 101.590(2)°, γ = 90°, V = 1676.45(7)Å^3^, Z = 4, D_calc_ = 1.942 gcm^‑3^, F(000) = 952, µ = 16.609 mm^‑1^, T = 120(1) K, θ_max_ = 66.722°, 23890 total reflections, 205238 with I_o_ > 2σ(I_o_), R_int_ = twin, 23890 data, 220 parameters, 0 restraints, GooF = 1.067, R = 0.0582 and wR = 0.1685 [I_o_ > 2σ(I_o_)], R = 0.0650 and wR = 0.1723 (all reflections), 1.254<d∆ρ < -1.322eÅ^‑3^.

**Comments on Checkcif file**: There are no A- and B-alerts

Supplementary Fig. 9 Crystal structure of **5-I** with the thermal displacement parameter at 50% probability level. Colour Key: purple = iodine, light orange = phosphorous, light green = fluorine, red = oxygen, blue = nitrogen, dark grey = carbon, and light cyan = hydrogen. Crystallization solvent = dichloroethane. Diffusion solvent = pentane.

**Crystal data for 5-I:** CCDC-2529416, C_12_H_14_F_6_IN_2_O_4_P, M = 522.12 gmol^‑1^, colourless block, 0.09 × 0.05 × 0.05 mm^3^, orthorhombic, space group *Cmmm*, a = 11.2664(13)Å, b = 7.5069(11)Å, c = 10.2297(14)Å, α= 90°, β = 90°, γ = 90°, V = 865.2(2) Å^3^, Z = 1, D_calc_ = 1.002 gcm^‑3^, F(000) = 254, µ = 8.116 mm^‑1^, T = 120(1) K, θ_max_ = 66.698°, 6087 total reflections, 462 with I_o_ > 2σ(I_o_), R_int_ = 0.0859, 465 data, 76 parameters, 0 restraints, GooF = 1.164, R = 0.0622 and wR = 0.1687 [I_o_ > 2σ(I_o_)], R = 0.0623 and wR = 0.1688 (all reflections), 1.889<d∆ρ < -0.743 eÅ^‑3^.

**Comments on Checkcif file**: There are no A-Alerts. There is a level B-alert. The level B-alert and our response are given below.

Alert level B

PLAT088_ALERT_3_B Poor Data / Parameter Ratio .................... 6.12 Note

**Author Response: This is due to disorder model and poor quality crystals**

Supplementary Fig. 10 Crystal structure of **5-Ia** with the thermal displacement parameter at 50% probability level. Colour Key: purple = iodine, pale pink = boron, light green = fluorine, red = oxygen, blue = nitrogen, dark grey = carbon, and light cyan = hydrogen. Crystallization solvent = dichloroethane. Diffusion solvent = pentane.

**Crystal data for 5-Ia:** CCDC-2529457, C_12_H_14_BF_4_IN_2_O_4_, M = 463.96 gmol^‑1^, colourless plate, 0.2 × 0.08 × 0.05 mm^3^, monoclinic, space group *C2/m*, a = 16.2552(13) Å, b = 4.0009(3) Å, c = 13.3454(11) Å, α= 90°, β = 98.418(7)°, γ = 90°, V = 858.57(12) Å^3^, Z = 1, D_calc_ = 0.897 gcm^‑3^, F(000) = 226, µ = 7.615 mm^‑1^, T = 120(1) K, θ_max_ = 66.732°, 5176 total reflections, 867 with I_o_ > 2σ(I_o_), R_int_ = 0.0422, 869 data, 105 parameters, 16 restraints, GooF = 1.147, R = 0.0379 and wR = 0.0973 [I_o_ > 2σ(I_o_)], R = 0.0379 and wR = 0.0973 (all reflections), 0.696<d∆ρ < -0.590 eÅ^‑3^.

**Comments on Checkcif file**: There are no A-Alerts. There is a level B-alert. The level B-alert and our response are given below.

Alert level B

PLAT049_ALERT_1_B Calculated Density Less Than 1.0 gcm-3 ......... 0.8973 Check

**Author Response: This is due to absorption artifacts**

Supplementary Fig. 11 Crystal structure of **6-I** with the thermal displacement parameter at 50% probability level. Colour Key: purple = iodine, red = oxygen, blue = nitrogen, dark grey = carbon, and light cyan = hydrogen. Crystallization solvent = dichloromethane. Diffusion solvent = pentane.

**Crystal data for 6-I:** CCDC-2529443, C_26_H_20_I_2_N_6_O_16_, M = 926.28 gmol^‑1^, colourless block, 0.04 × 0.03 × 0.02 mm^3^, triclinic, space group *P-1*, a = 9.0796(8) Å, b = 9.9971(9) Å, c = 10.3215(8) Å, α= 77.475(7) °, β = 69.198(8) °, γ = 66.421(9) °, V = 799.62(14) Å^3^, Z = 1, D_calc_ = 1.924 gcm^‑3^, F(000) = 452, µ = 16.214 mm^‑1^, T = 120(1) K, θ_max_ = 66.749 °, 8060 total reflections, 1906 with I_o_ > 2σ(I_o_), R_int_ = 0.0636, 2793 data, 230 parameters, 16 restraints, GooF = 1.024, R = 0.0609 and wR = 0.1602 [I_o_ > 2σ(I_o_)], R = 0.0889 and wR = 0.1780 (all reflections), 2.760 <d∆ρ < -2.077 eÅ^‑3^.

**Comments on Checkcif file**: There are no A-Alerts. There is a level B-alert. The level B-alert and our response are given below.

Alert level B

PLAT971_ALERT_2_B Check Calcd Resid. Dens. 0.96Ang From I2	2.62 eA-3

**Author Response: This is due to absorption artifacts**

Supplementary Fig. 12 Crystal structure of **7-I** with the thermal displacement parameter at 50% probability level. Colour Key: purple = iodine, brown = bromine, light orange = phosphorous, light green = fluorine, red = oxygen, blue = nitrogen, dark grey = carbon, and light cyan = hydrogen. Crystallization solvent = dichloromethane. Diffusion solvent = pentane.

**Crystal data for 7-I:** CCDC-2529458, C_10_H_8_Br_2_F_6_IN_2_O_2_P, M = 619.87 gmol^‑1^, yellow block, 0.21× 0.15× 0.05mm^3^, triclinic, space group *P-1*, a = 6.1053(2)Å, b = 6.7413(2) Å, c = 10.6133(3)Å, α= 72.481(3)°, β = 86.681(2)°, γ = 79.828(3)°, V = 410.01(2)Å^3^, Z = 1, D_calc_ = 2.511gcm^‑3^, F(000) = 290, µ = 6.993 mm^‑1^, T = 120(1) K, θ_max_ = 25.241°, 5040 total reflections, 1379 with I_o_ > 2σ(I_o_), R_int_ = 0.0290, 1479 data, 112 parameters, 0 restraints, GooF = 1.072, R = 0.0214 and wR = 0.0520 [I_o_ > 2σ(I_o_)], R = 0.0232 and wR = 0.0529 (all reflections), 0.413<d∆ρ < -0.502eÅ^‑3^.

**Comments on Checkcif file**: There are no A- and B-alerts

**4.2 Silver(I) complexes of pyridines**

Supplementary Fig. 13 Crystal structure of {**(3MePy)_2_Ag}PF_6_** (3MePy = 3-methylpyridine) with the thermal displacement parameter at 50% probability level. Colour Key: ash white = silver, light orange = phosphorous, light green = fluorine, blue = nitrogen, dark grey = carbon, and white = hydrogen. Crystallization solvent = dichloromethane, diffusion solvent = pentane.

**Crystal data for** {**(3MePy)_2_Ag}PF_6_:** CCDC-2529424, C_12_H_14_AgF_6_N_2_P, M = 439.09 gmol^‑1^, colourless block, 0.09× 0.08× 0.06 mm^3^, monoclinic, space group *P2_1_/n*, a = 8.23430(10)Å, b = 17.5483(2)Å, c = 10.91920(10)Å, α= 90°, β = 103.7290(10)°, γ = 90°, V = 1532.72(3)Å^3^, Z = 4, D_calc_ = 1.903 gcm^‑3^, F(000) = 864, µ = 12.136 mm^‑1^, T = 120(1) K, θ_max_ = 66.744°, 20311 total reflections, 2647 with I_o_ > 2σ(I_o_), R_int_ = 0.0241, 2713 data, 201 parameters, 0 restraints, GooF = 1.131, R = 0.0166 and wR = 0.0395 [I_o_ > 2σ(I_o_)], R = 0.0173 and wR = 0.0398 (all reflections), 0.360<d∆ρ < -0.444eÅ^‑3^.

**Comments on Checkcif file**: There are no A- and B-alerts

Supplementary Fig. 14 Crystal structure of {**(3MePy)_2_Ag}PF_6__1** (3MePy = 3-methylpyridine) with the thermal displacement parameter at 50% probability level. Colour Key: ash white = silver, light orange = phosphorous, light green = fluorine, blue = nitrogen, dark grey = carbon, and white = hydrogen. Crystallization solvent = dichloroethane. Diffusion solvent = pentane.

**Crystal data for** {**(3MePy)_2_Ag}PF_6__1:** CCDC-2529453, C_12_H_14_AgF_6_N_2_P, M = 439.09 gmol^‑1^, colourless block, 0.11× 0.02× 0.02 mm^3^, monoclinic, space group *C2/c*, a = 27.4314(10)Å, b = 8.4552(3)Å, c = 13.7553(5)Å, α= 90°, β = 92.060(3)°, γ = 90°, V = 3188.3(2)Å^3^, Z = 8, D_calc_ = 1.830 gcm^‑3^, F(000) = 1728, µ = 11.668 mm^‑1^, T = 120(1) K, θ_max_ = 66.744°, 12902 total reflections, 2326 with I_o_ > 2σ(I_o_), R_int_ = 0.0891, 2794 data, 205 parameters, 0 restraints, GooF = 1.096, R = 0.0698 and wR = 0.1941 [I_o_ > 2σ(I_o_)], R = 0.0897 and wR = 0.2400 (all reflections), 1.030<d∆ρ < -2.573eÅ^‑3^.

**Comments on Checkcif file**: There are no A-Alerts. There is a level B-alert. The level B-alert and our response are given below.

Alert level B

PLAT972_ALERT_2_B Check Calcd Resid. Dens. 0.86Ang From Ag1	-3.16 eA-3

**Author Response: This is due to the absorption artifacts**

Supplementary Fig. 15 Crystal structure of {**(2MeOPy)_2_Ag}PF_6_** (2MeOPy = 2-methoxypyridine) with the thermal displacement parameter at 50% probability level. Colour Key: ash white = silver, light orange = phosphorous, light green = fluorine, red = oxygen, blue = nitrogen, dark grey = carbon, and white = hydrogen. Crystallization solvent = dichloroethane, diffusion solvent = pentane.

**Crystal data for** {**(2MeOPy)_2_Ag}PF_6_:** CCDC-2529436, C_12_H_14_AgF_6_N_2_O_2_P, M = 471.09 gmol^‑1^, colourless block, 0.15 × 0.13 × 0.1 mm^3^, triclinic, space group *P -1*, a = 6.84720(10) Å, b = 10.96500(10) Å, c = 11.0657(2) Å, α= 77.4930(10) °, β = 85.3190(10) °, γ = 84.1500(10) °, V = 805.35(2) Å^3^, Z = 2, D_calc_ = 1.943 gcm^‑3^, F(000) = 464, µ = 11.699 mm^‑1^, T = 120(1) K, θ_max_ = 66.739 °, 15762 total reflections, 2809 with I_o_ > 2σ(I_o_), R_int_ = 0.0364, 2845 data, 219 parameters, 0 restraints, GooF = 1.079, R = 0.0273 and wR = 0.0701 [I_o_ > 2σ(I_o_)], R = 0.0276 and wR = 0.0704 (all reflections), 0.950 <d∆ρ < -0.459 eÅ^‑3^.

**Comments on Checkcif file**: There are no A- and B-alerts

Supplementary Fig. 16 Crystal structure of {**(2MeOPy)_2_Ag}PF_6__1** (2MeOPy = 2-methoxypyridine) with the thermal displacement parameter at 50% probability level. Colour Key: ash white = silver, light orange = phosphorous, light green = fluorine, red = oxygen, blue = nitrogen, dark grey = carbon, and white = hydrogen. Crystallization solvent = dichloromethane, diffusion solvent = pentane.

**Crystal data for** {**(2MeOPy)_2_Ag}PF_6__1:** CCDC-2529413, C_12_H_14_AgF_6_N_2_O_2_P, M = 471.09 gmol^‑1^, colourless block, 0.07× 0.06× 0.02mm^3^, triclinic, space group *P -1*, a = 7.9085(3)Å, b = 8.0138(2)Å, c = 13.5793(4)Å, α= 79.069(3)°, β = 84.651(3)°, γ = 76.774(3)°, V = 821.47(5)Å^3^, Z = 2, D_calc_ = 1.905 gcm^‑3^, F(000) = 464, µ = 11.470mm^‑1^, T = 120(1) K, θ_max_ = 66.732°, 9293 total reflections, 2810 with I_o_ > 2σ(I_o_), R_int_ = 0.0298, 2916 data, 219 parameters, 0 restraints, GooF = 1.085, R = 0.0252and wR = 0.0633 [I_o_ > 2σ(I_o_)], R = 0.0262 and wR = 0.0637 (all reflections), 0.753<d∆ρ < -0.595eÅ^‑3^.

**Comments on Checkcif file**: There are no A- and B-alerts

Supplementary Fig. 17 Crystal structure of {**(4MeOPy)_2_Ag}PF_6_** (4MeOPy = 4-methoxypyridine) with the thermal displacement parameter at 50% probability level. Colour Key: ash white = silver, light orange = phosphorous, light green = fluorine, red = oxygen, blue = nitrogen, dark grey = carbon, and white = hydrogen. Crystallization solvent = acetone, diffusion solvent = pentane. Acetone molecules were omitted for viewing clarity.

**Crystal data for** {**(4MeOPy)_2_Ag}PF_6_:** CCDC- 2529431, C_15_H_20_AgF_6_N_2_O_3_P, M = 529.17 gmol^‑1^, colourless block, 0.16 × 0.08 × 0.07 mm^3^, monoclinic, space group *P2_1_/n*, a = 6.62510(10) Å, b = 16.3459(2) Å, c = 18.1122(3) Å, α= 90 °, β = 92.7440(10)°, γ = 90 °, V = 1959.18(5) Å^3^, Z = 4, D_calc_ = 1.794 gcm^‑3^, F(000) = 1056, µ = 9.736 mm^‑1^, T = 120(1) K, θ_max_ = 66.736°, 35010 total reflections, 29794 with I_o_ > 2σ(I_o_), R_int_ = twin, 35010 data, 258 parameters, 0 restraints, GooF = 1.103, R = 0.0474 and wR = 0.1663 [I_o_ > 2σ(I_o_)], R = 0.0636 and wR = 0.2314 (all reflections), 1.381 <d∆ρ < -0.608 eÅ^‑3^.

**Comments on Checkcif file**: There are no A- and B-alerts

Supplementary Fig. 18 Crystal structure of {**(3BrPy)_2_Ag}PF_6_** (3BrPy = 3-bromopyridine) with the thermal displacement parameter at 50% probability level. Colour Key: ash white = silver, brown = bromine, light orange = phosphorous, light green = fluorine, blue = nitrogen, dark grey = carbon, and white = hydrogen. Crystallization solvent = dichloroethane, diffusion solvent = pentane. Disordered parts were omitted for viewing clarity.

**Crystal data for** {**(3BrPy)_2_Ag}PF_6_:** CCDC-2529435, C_40_H_32_Ag_4_Br_8_F_24_N_8_P_4_, M = 2275.37 gmol^‑1^, colourless block, 0.15 × 0.12 × 0.09 mm^3^, tetragonal, space group *I4_1_/amd*, a = 15.0686(2) Å, b = 15.0686(2) Å, c = 13.8331(3) Å, α= 90 °, β = 90 °, γ = 90 °, V = 3140.98(11) Å^3^, Z = 2, D_calc_ = 2.406 gcm^‑3^, F(000) = 2144, µ = 17.808 mm^‑1^, T = 120(1) K, θ_max_ = 66.547°, 13658 total reflections, 739 with I_o_ > 2σ(I_o_), R_int_ = 0.0356, 758 data, 74 parameters, 18 restraints, GooF = 1.106, R = 0.0552 and wR = 0.1408 [I_o_ > 2σ(I_o_)], R = 0.0561 and wR = 0.1414 (all reflections), 0.846 <d∆ρ < -0.989 eÅ^‑3^.

**Comments on Checkcif file**: There are no A- and B-alerts

**4.3 Iodine(I) complexes of pyridine**

Supplementary Fig. 19 Crystal structure of {**(3MePy)_2_I}PF_6_** (3MePy = 3-methylpyridine) with the thermal displacement parameter at 50% probability level. Colour Key: purple = iodine, light orange = phosphorous, light green = fluorine, blue = nitrogen, dark grey = carbon, and white = hydrogen. Crystallization solvent = dichloroethane, diffusion solvent = pentane.

**Crystal data for** {**(3MePy)_2_I}PF_6_:** CCDC-2529455, C_12_H_14_F_6_IN_2_P, M = 458.12 gmol^‑1^, colourless block, 0.14 × 0.12× 0.05 mm^3^, triclinic, space group *P-1*, a = 6.9537(2) Å, b = 7.84620(10) Å, c = 8.54310(10) Å, α= 104.7200(10)°, β = 96.437(2)°, γ = 114.749(2)°, V = 396.539(15) Å^3^, Z = 1, D_calc_ = 1.918 gcm^‑3^, F(000) = 222, µ = 17.401 mm^‑1^, T = 120(1) K, θ_max_ = 66.717 °, 7683 total reflections, 1412 with I_o_ > 2σ(I_o_), R_int_ = 0.0282, 1412 data, 105 parameters, 0 restraints, GooF = 1.173, R = 0.0757 and wR = 0.2228 [I_o_ > 2σ(I_o_)], R = 0.0757 and wR = 0.2228 (all reflections), 6.764 <d∆ρ < -1.262 eÅ^‑3^.

**Comments on Checkcif file**: There are no A-Alerts. There is a level B-alert. The level B-alert and our response are given below.

Alert level B

PLAT094_ALERT_2_B Ratio of Maximum / Minimum Residual Density .... 5.36 Report

**Author Response: This is due to the absorption artifacts**

PLAT097_ALERT_2_B Large Reported Max. (Positive) Residual Density	6.76 eA-3

**Author Response: This is due to the absorption artifacts**

Supplementary Fig. 20 Crystal structure of {**(2MeOPy)_2_I}PF_6_** (2MeOPy = 2-methoxypyridine) with the thermal displacement parameter at 50% probability level. Colour Key: purple = iodine, light orange = phosphorous, light green = fluorine, blue = nitrogen, red = oxygen, dark grey = carbon, and white = hydrogen. Crystallization solvent = dichloroethane, diffusion solvent = pentane.

**Crystal data for** {**(2MeOPy)_2_I}PF_6_:** CCDC- 2531719, C_12_H_14_F_6_IN_2_O_2_P, M = 490.12 gmol^‑1^, colourless block, 0.1× 0.08× 0.04 mm^3^, triclinic, space group *P-1*, a = 7.9183(2)Å, b = 8.0052(2)Å, c = 13.5785(3)Å, α= 79.031(2)°, β = 84.720(2)°, γ = 76.806(2) °, V = 821.61(4) Å^3^, Z = 2, D_calc_ = 1.981gcm^‑3^, F(000) = 476, µ = 16.945 mm^‑1^, T = 120(1) K, θ_max_ = 66.747°, 15995 total reflections, 2774 with I_o_ > 2σ(I_o_), R_int_ = 0.0471, 2915 data, 219 parameters, 0 restraints, GooF = 1.138, R = 0.0247 and wR = 0.0613 [I_o_ > 2σ(I_o_)], R = 0.0265 and wR = 0.0628 (all reflections), 0.586<d∆ρ < -0.836eÅ^‑3^.

**Comments on Checkcif file**: There are no A- and B-alerts

Supplementary Fig. 21 Crystal structure of {**(4MeOPy)_2_I}PF_6_** (4MeOPy = 4-methoxypyridine) with the thermal displacement parameter at 50% probability level. Colour Key: purple = iodine, light orange = phosphorous, light green = fluorine, blue = nitrogen, red = oxygen, dark grey = carbon, and white = hydrogen. Crystallization solvent = dichloroethane, diffusion solvent = pentane.

**Crystal data for** {**(4MeOPy)_2_I}PF_6_:** CCDC-2529432, C_12_H_14_F_6_IN_2_O_2_P, M = 490.12 gmol^‑1^, colourless plate, 0.22 × 0.13 × 0.05 mm^3^, triclinic, space group *P-1*, a = 6.8259(4) Å, b = 9.6260(6) Å, c = 15.4167(7) Å, α= 74.536(5) °, β = 80.288(5) °, γ = 74.664(5) °, V = 936.27(10) Å^3^, Z = 2, D_calc_ = 1.739 gcm^‑3^, F(000) = 476, µ = 14.870 mm^‑1^, T = 120(1) K, θ_max_ = 66.738 °, 10423 total reflections, 8616 with I_o_ > 2σ(I_o_), R_int_ = twin, 10423 data, 220 parameters, 0 restraints, GooF = 1.063, R = 0.0722 and wR = 0.1913 [I_o_ > 2σ(I_o_)], R = 0.0838 and wR = 0.2004 (all reflections), 1.669 <d∆ρ < -1.476 eÅ^‑3^.

**Comments on Checkcif file**: There are no A- and B-alerts

Supplementary Fig. 22 Crystal structure of {**(3BrPy)_2_I}PF_6_** (3BrPy = 3-bromopyridine) with the thermal displacement parameter at 50% probability level. Colour Key: purple = iodine, brown = bromine, light orange = phosphorous, light green = fluorine, blue = nitrogen, dark grey = carbon, and light cyan = hydrogen. Crystallization solvent = dichloroethane, diffusion solvent = pentane.

**Crystal data for** {**(3BrPy)_2_I}PF_6_:** CCDC-2529440, C_10_H_8_Br_2_F_6_IN_2_P, M = 587.87gmol^‑1^, colourless block, 0.09× 0.06× 0.05 mm^3^, orthorhombic, space group *Cmc2_1_*, a = 6.91399(9)Å, b = 14.25588(17) Å, c = 15.88265(18)Å, α= 90°, β = 90°, γ = 90°, V = 1565.48(3) Å^3^, Z = 4, D_calc_ = 2.494gcm^‑3^, F(000) = 1096, µ = 23.614 mm^‑1^, T = 120(1) K, θ_max_ = 66.723°, 6284 total reflections, 1421 with I_o_ > 2σ(I_o_), R_int_ = 0.0247, 1427 data, 127 parameters, 1 restraints, GooF = 1.077, R = 0.0146 and wR = 0.0369 [I_o_ > 2σ(I_o_)], R = 0.0147 and wR = 0.0369 (all reflections), 0.283<d∆ρ < -0.366eÅ^‑3^.

**Comments on Checkcif file**: There are no A- and B-alerts

**4.4 Silver(I) complexes of pyridine *N*-oxides**

Supplementary Fig. 23 Asymmetric unit of **1-Ag** with the thermal displacement parameter at 50% probability level. Colour Key: ash white = silver, light orange = phosphorous, light green = fluorine, red = oxygen, blue = nitrogen, dark grey = carbon, and light cyan = hydrogen. Crystallization solvent = dichloroethane, diffusion solvent = pentane. For full polymeric structure see Figure S26.

**Crystal data for 1-Ag:** CCDC-2529418, C_10_H_10_AgF_6_N_2_O_2_P, M = 443.04gmol^‑1^, colourless block, 0.23× 0.055 × 0.048mm^3^, monoclinic, space group *I2/m*, a = 6.89400(10)Å, b = 11.8946(2)Å, c = 19.0416(3)Å, α= 90°, β = 91.4850(10)°, γ = 90°, V = 1560.91(4)Å^3^, Z = 4, D_calc_ = 1.885 gcm^‑3^, F(000) = 864, µ = 12.026 mm^‑1^, T = 120(1) K, θ_max_ = 76.522°, 7601 total reflections, 1613 with I_o_ > 2σ(I_o_), R_int_ = 0.0217, 1704 data, 111 parameters, 0 restraints, GooF = 1.083, R = 0.0208 and wR = 0.0525 [I_o_ > 2σ(I_o_)], R = 0.0222 and wR = 0.0533 (all reflections), 0.587<d∆ρ < -0.498eÅ^‑3^.

**Comments on Checkcif file**: There are no A- and B-alerts

Supplementary Fig. 24 Asymmetric unit of **2-Ag** with the thermal displacement parameter at 50% probability level. Colour Key: ash white = silver, light orange = phosphorous, light green = fluorine, red = oxygen, blue = nitrogen, dark grey = carbon, and light cyan = hydrogen. Crystallization solvent = dichloroethane, diffusion solvent = pentane. For full polymeric structure see Figure S26.

**Crystal data for 2-Ag:** CCDC-2529423, C_12_H_14_AgF_6_N_2_O_2_P, M = 471.09gmol^‑1^, colourless block, 0.22× 0.05× 0.03mm^3^, monoclinic, space group *I2/a*, a = 10.2750(3)Å, b = 23.2499(4)Å, c = 13.9582(3)Å, α= 90°, β = 110.592(3)°, γ = 90°, V = 3121.47(14)Å^3^, Z = 8, D_calc_ = 2.005gcm^‑3^, F(000) = 1856, µ = 12.074mm^‑1^, T = 120(1) K, θ_max_ = 66.732°, 14608 total reflections, 2472 with I_o_ > 2σ(I_o_), R_int_ = 0.0531, 2772 data, 221 parameters, 0 restraints, GooF = 1.063, R = 0.0532 and wR = 0.1377 [I_o_ > 2σ(I_o_)], R = 0.0580 and wR = 0.1417 (all reflections), 2.134<d∆ρ < -1.292eÅ^‑3^.

**Comments on Checkcif file**: There are no A- and B-alerts

Supplementary Fig. 25 Asymmetric unit of **4-Ag** with the thermal displacement parameter at 50% probability level. Colour Key: ash white = silver, light orange = phosphorous, light green = fluorine, blue = nitrogen, red = oxygen, dark grey = carbon, and light cyan = hydrogen. Crystallization solvent = dichloroethane, diffusion solvent = pentane. For full polymeric structure see Figure S26.

**Crystal data for 4-Ag:** CCDC- 2529419, C_48_H_56_Ag_3_F_18_N_8_O_8_P_3_, M = 1631.52 gmol^‑1^, colourless block, 0.1554× 0.0951× 0.0477mm^3^, triclinic, space group *P-1*, a = 7.1101(2)Å, b = 13.7688(5)Å, c = 16.4199(5)Å, α= 66.882(3)°, β = 89.088(2)°, γ = 86.507(3)°, V = 1475.58(9)Å^3^, Z = 1, D_calc_ = 1.836gcm^‑3^, F(000) = 812, µ = 9.716 mm^‑1^, T = 123(1)K, θ_max_ = 66.744°, 16697 total reflections, 4575 with I_o_ > 2σ(I_o_), R_int_ = 0.0463, 5221 data, 404 parameters, 0 restraints, GooF = 1.032, R = 0.0300 and wR = 0.0748 [I_o_ > 2σ(I_o_)], R = 0.0358 and wR = 0.0803 (all reflections), 0.629<d∆ρ < -0.823eÅ^‑3^.

**Comments on Checkcif file**: There are no A- and B-alerts

Supplementary Fig. 26 Partial packing structures of (a) **1-Ag** (b) **2-Ag** (c) **4-Ag** to show denticity of *N*-oxide oxygen and coordination number of silver(I). Selected atoms are shown in wireframe for viewing clarity. Anions were omitted for viewing clarity.

Supplementary Fig. 27 Asymmetric unit of **1-Ag_1** with the thermal displacement parameter at 50% probability level. Colour Key: ash white = silver, light orange = phosphorous, light green = fluorine, red = oxygen, blue = nitrogen, dark grey = carbon, and light cyan = hydrogen. Crystallization solvent = Acetonitrile. Disordered parts were omitted for clarity. For full polymeric structure see Figure S31.

**Crystal data for 1-Ag_1:** CCDC-2529428, C_40_H_40_Ag_3_F_18_N_8_O_8_P_3_, M = 1519.32gmol^‑1^, colourless block, 0.14× 0.12× 0.1 mm^3^, monoclinic, space group *P2/c*, a = 14.0591(2)Å, b = 13.7214(2)Å, c = 13.5878(2)Å, α= 90°, β = 104.696(2)°, γ = 90°, V = 2535.48(7)Å^3^, Z = 2, D_calc_ = 1.990gcm^‑3^, F(000) = 1496, µ = 11.252mm^‑1^, T = 120(1) K, θ_max_ = 66.743°, 21236 total reflections, 4161 with I_o_ > 2σ(I_o_), R_int_ = 0.0315, 4497 data, 507 parameters, 222 restraints, GooF = 1.030, R = 0.0313 and wR = 0.0766 [I_o_ > 2σ(I_o_)], R = 0.0342 and wR = 0.0781 (all reflections), 0.810<d∆ρ < -1.139eÅ^‑3^.

**Comments on Checkcif file**: There are no A- and B-alerts

Supplementary Fig. 28 Asymmetric unit of **3-Ag** with the thermal displacement parameter at 50% probability level. Colour Key: ash white = silver, light orange = phosphorous, light green = fluorine, red = oxygen, blue = nitrogen, dark grey = carbon, and light cyan = hydrogen. Crystallization solvent = Acetonitrile. For full polymeric structure see Figure S31.

**Crystal data for 3-Ag:** CCDC-2529439, C_18_H_21_AgF_6_N_3_O_3_P, M = 579.46 gmol^‑1^, colourless block, 0.13× 0.05× 0.03mm^3^, hexagonal, space group *P-62c*, a = 14.0047(2)Å, b = 14.0047(2)Å, c = 6.30950(10)Å, α= 90°, β = 90°, γ = 120°, V = 1071.70(4) Å^3^, Z = 2, D_calc_ = 1.796gcm^‑3^, F(000) = 579, µ = 8.978mm^‑1^, T = 120(1) K, θ_max_ = 66.532°, 11305 total reflections, 668 with I_o_ > 2σ(I_o_), R_int_ = 0.0502, 702 data, 67 parameters, 19 restraints, GooF = 1.193, R = 0.0423 and wR = 0.1140 [I_o_ > 2σ(I_o_)], R = 0.0442 and wR = 0.1177 (all reflections), 0.916<d∆ρ < -0.671 eÅ^‑3^.

**Comments on Checkcif file**: There are no A-Alerts. There is a level B-alert. The level B-alert and our response are given below.

Alert level B

PLAT090_ALERT_3_B Poor Data / Parameter Ratio (Zmax > 18) ........ 5.94 Note

**Author Response: Due to unresolved disorder**

PLAT230_ALERT_2_B Hirshfeld Test Diff for	O1	--N1	. 11.4 s.u.

**Author Response: Due to unresolved disorder**

PLAT342_ALERT_3_B Low Bond Precision on C-C Bonds ............... 0.032 Ang.

**Author Response: Due to unresolved disorder**

PLAT987_ALERT_1_B The Flack x is >> 0 - Do a BASF/TWIN Refinement	Please Check

**Author Response: Twin refinement done**

Supplementary Fig. 29 Asymmetric unit of **4-Ag_1** with the thermal displacement parameter at 50% probability level. Colour Key: ash white = silver, light light orange = phosphorous, light green = fluorine, red = oxygen, blue = nitrogen, dark grey = carbon, and light cyan = hydrogen. Crystallization solvent = Acetonitrile. For full polymeric structure see Figure S32.

**Crystal data for 4-Ag_1:** CCDC-2529437, C_18_H_21_AgF_6_N_3_O_3_P, M = 579.93gmol^‑1^, colourless block, 0.184× 0.124× 0.057mm^3^, hexagonal, space group *P6_3_/m*, a = 14.2321(2)Å, b = 14.2321(2)Å, c = 6.17290(10)Å, α= 90°, β = 90°, γ = 120°, V = 1082.82(4)Å^3^, Z = 2, D_calc_ = 1.779gcm^‑3^, F(000) = 580, µ = 8.889 mm^‑1^, T = 120(1) K, θ_max_ = 66.505°, 7967 total reflections, 694 with I_o_ > 2σ(I_o_), R_int_ = 0.0227, 706 data, 81 parameters, 26 restraints, GooF = 1.088, R = 0.0144 and wR = 0.0389 [I_o_ > 2σ(I_o_)], R = 0.0147 and wR = 0.0390 (all reflections), 0.264<d∆ρ < -0.312eÅ^‑3^.

**Comments on Checkcif file**: There are no A- and B-alerts

Supplementary Fig. 30 Asymmetric unit of **5-Ag** with the thermal displacement parameter at 50% probability level. Colour Key: ash white = silver, light orange = phosphorous, light green = fluorine, red = oxygen, blue = nitrogen, dark grey = carbon, and light cyan = hydrogen. Crystallization solvent = Acetonitrile. For full polymeric structure see Figure S32.

**Crystal data for 5-Ag:** CCDC-2529438, C_12_H_14_AgBF_4_N_2_O_4_, M = 444.93 gmol^‑1^, colourless block, 0.1 × 0.04 × 0.03 mm^3^, triclinic, space group *P-1*, a = 6.6159(6) Å, b = 10.3381(7) Å, c = 12.8740(12) Å, α= 110.258(7)°, β = 96.572(8) °, γ = 105.169(7) °, V = 776.57(12) Å^3^, Z = 2, D_calc_ = 1.903 gcm^‑3^, F(000) = 440, µ = 11.044 mm^‑1^, T = 120(1) K, θ_max_ = 66.750 °, 10055 total reflections, 8415 with I_o_ > 2σ(I_o_), R_int_ = twin, 10055 data, 220 parameters, 0 restraints, GooF = 1.210, R = 0.0557 and wR = 0.1813 [I_o_ > 2σ(I_o_)], R = 0.0626 and wR = 0.1854 (all reflections), 3.257 <d∆ρ < -3.557 eÅ^‑3^.

**Comments on Checkcif file**: There are no A- and B-alerts

Supplementary Fig. 31 Partial packing structures of (a) **1-Ag_1** and (b) **3-Ag** displaying denticity of *N*-oxide oxygen and coordination number of silver(I). Selected atoms are shown in wireframe for viewing clarity. Anions were omitted for viewing clarity.

Supplementary Fig. 32 Partial packing structures of (a) **4-Ag_1** and (b) **5-Ag** displaying denticity of *N*-oxide oxygen and coordination number of silver(I). Selected atoms are shown in wireframe for viewing clarity. Anions were omitted for viewing clarity.

Supplementary Fig. 33 Crystal structure of {**(4)_2_CH_2_}(OTf)_2_** with the thermal displacement parameter at 50% probability level. Colour Key: yellow = sulfur, light green = fluorine, red = oxygen, blue = nitrogen, dark grey = carbon, and light cyan = hydrogen. Crystallization solvent = dichloromethane, diffusion solvent = pentane.

**Crystal data for** {**(4)_2_CH_2_}(OTf)_2_:** CCDC- 2529420, C_15_H_16_F_6_N_2_O_8_S_2_, M = 530.42 gmol^‑1^, colourless block, 0.14× 0.1× 0.07mm^3^, triclinic, space group *P-1*, a = 8.3290(3)Å, b = 10.0775(3)Å, c = 14.1369(4)Å, α= 77.762(2)°, β = 76.844(3)°, γ = 68.137(3)°, V = 1061.66(6)Å^3^, Z = 2, D_calc_ = 1.659gcm^‑3^, F(000) = 540, µ = 3.215 mm^‑1^, T = 120(1) K, θ_max_ = 66.747°, 14297 total reflections, 3460 with I_o_ > 2σ(I_o_), R_int_ = 0.0329, 3759 data, 300 parameters, 0 restraints, GooF = 1.055, R = 0.0314 and wR = 0.0814 [I_o_ > 2σ(I_o_)], R = 0.0340 and wR = 0.0831 (all reflections), 0.347<d∆ρ < -0.422eÅ^‑3^.

**Comments on Checkcif file**: There are no A- and B-alerts

**4.5 Hydrogen-bonded complexes of pyridine *N*-oxides**

Supplementary Fig. 34 Crystal structure of **(TMNOH)CH_3_SO_3_** (TMNO = trimethylamine *N*-oxide) with the thermal displacement parameter at 50% probability level. Colour Key: yellow = sulphur, blue = nitrogen, red = oxygen, dark grey = carbon, and light cyan = hydrogen. Crystallization solvent = dichloroethane.

**Crystal data for (TMNOH)CH_3_SO_3_:** CCDC-2529446, C_4_H_13_NO_4_S, M = 171.21 gmol^‑1^, colourless block, 0.19 × 0.15 × 0.1 mm^3^, orthorhombic, space group *Pbca*, a = 11.35337(14) Å, b = 11.29617(14) Å, c = 12.24391(15) Å, α= 90°, β = 90 °, γ = 90°, V = 1570.28(3) Å^3^, Z = 8, D_calc_ = 1.448 gcm^‑3^, F(000) = 736, µ = 3.427 mm^‑1^, T = 120(1) K, θ_max_ = 66.721 °, 12345 total reflections, 1368 with I_o_ > 2σ(I_o_), R_int_ = 0.0205, 1383 data, 96 parameters, 0 restraints, GooF = 1.060, R = 0.0246 and wR = 0.0644 [I_o_ > 2σ(I_o_)], R = 0.0248 and wR = 0.0645 (all reflections), 0.319 <d∆ρ < -0.440 eÅ^‑3^.

**Comments on Checkcif file**: There are no A- and B-alerts

Supplementary Fig. 35 Crystal structure of {**(2)_2_H}SbF_6_** with the thermal displacement parameter at 50% probability level. Colour Key: light pink = antimony, light green = fluorine, blue = nitrogen, red = oxygen, dark grey = carbon, and light cyan = hydrogen. Crystallization solvent = dichloroethane. Diffusion solvent = hexane.

**Crystal data for** {**(2)_2_H}SbF_6_:** CCDC-2529444, C_12_H1_5_F_6_N_2_O_2_Sb, M = 455.01 gmol^‑1^, colourless block, 0.21 × 0.1 × 0.07 mm^3^, tetragonal, space group *I4_1_/a*, a = 8.72230(10) Å, b = 8.72230(10) Å, c = 42.2021(5) Å, α= 90 °, β = 90 °, γ = 90 °, V = 3210.67(8) Å^3^, Z = 8, D_calc_ = 1.883 gcm^‑3^, F(000) = 1776, µ = 14.335 mm^‑1^, T = 120(1) K, θ_max_ = 66.700°, 25304 total reflections, 1403 with I_o_ > 2σ(I_o_), R_int_ = 0.0558, 1434 data, 108 parameters, 0 restraints, GooF = 1.088, R = 0.0246 and wR = 0.0601 [I_o_ > 2σ(I_o_)], R = 0.0252 and wR = 0.0605 (all reflections), 0.547 <d∆ρ < -0.702 eÅ^‑3^.

**Comments on Checkcif file**: There are no A- and B-alerts

Supplementary Fig. 36 Crystal structure of {**(2)_2_H}PF_6_** with the thermal displacement parameter at 50% probability level. Colour Key: light orange = phosphorous, light green = fluorine, blue = nitrogen, red = oxygen, dark grey = carbon, and light cyan = hydrogen. Crystallization solvent = dichloroethane. Diffusion = pentane.

**Crystal data for** {**(2)_2_H}PF_6_:** CCDC-2529417, C_12_H_15_F_6_N_2_O_2_P, M = 364.23 gmol^‑1^, colourless block, 0.091 × 0.08 × 0.08 mm^3^, triclinic, space group *P-1*, a = 8.1880(6)Å, b = 10.3930(9) Å, c = 10.7393(10) Å, α= 64.211(9) °, β = 71.890(8) °, γ = 70.718(7) °, V = 761.54(13)Å^3^, Z = 2, D_calc_ = 1.588 gcm^‑3^, F(000) = 372, µ = 2.340 mm^‑1^, T = 120(1) K, θ_max_ = 66.733°, 9425 total reflections, 2177 with I_o_ > 2σ(I_o_), R_int_ = 0.0439, 2696 data, 214 parameters, 0 restraints, GooF = 1.107, R = 0.0605 and wR = 0.1681 [I_o_ > 2σ(I_o_)], R = 0.0719 and wR = 0.1762 (all reflections), 0.611<d∆ρ < -0.387 eÅ^‑3^.

**Comments on Checkcif file**: There are no A- and B-alerts

Supplementary Fig. 37 Crystal structure of **(2H)PF_6_** with the thermal displacement parameter at 50% probability level. Colour Key: light orange = phosphorous, light green = fluorine, blue = nitrogen, red = oxygen, dark grey = carbon, and light cyan = hydrogen. Crystallization solvent = dichloroethane. Diffusion = pentane. This hydrogen-bonded structure was collected from a different batch.

**Crystal data for (2H)PF_6_:** CCDC-2529452, C_12_H_16_F_6_N_2_O_2_P, M = 365.24 gmol^‑1^, colourless block, 0.08 × 0.08 × 0.06 mm^3^, monoclinic, space group *P2_1_/c*, a = 6.64690(10)Å, b = 13.0906(2) Å, c = 8.47140(10) Å, α= 90°, β = 96.8480(10) °, γ = 90°, V = 731.854(18)Å^3^, Z = 2, D_calc_ = 1.657 gcm^‑3^, F(000) = 374, µ = 2.435 mm^‑1^, T = 120(1) K, θ_max_ = 66.747°, 8725 total reflections, 1190 with I_o_ > 2σ(I_o_), R_int_ = 0.0443, 1294 data, 111 parameters, 0 restraints, GooF = 1.060, R = 0.0308 and wR = 0.0850 [I_o_ > 2σ(I_o_)], R = 0.0331 and wR = 0.0866 (all reflections), 0.207<d∆ρ < -0.531 eÅ^‑3^.

**Comments on Checkcif file**: There are no A- and B-alerts

Supplementary Fig. 38 Crystal structure of {**(3H)3}ClO_4_** with the thermal displacement parameter at 50% probability level. Colour Key: green = chlorine, blue = nitrogen, red = oxygen, dark grey = carbon, and light cyan = hydrogen. Crystallization solvent = dichloromethane. Diffusion = pentane. The same structure was obtained with dichloroethane and hexane combinations.

**Crystal data for** {**(3H)3}ClO_4_:** CCDC-2529425, C_12_H_15_ClN_2_O_6_, M = 318.71gmol^‑1^, colourless block, 0.111× 0.089× 0.032mm^3^, monoclinic, space group *P2_1_/c*, a = 14.0570(3)Å, b = 6.87690(10) Å, c = 15.6904(3)Å, α= 90°, β = 109.468(2)°, γ = 90°, V = 1430.05(5) Å^3^, Z = 4, D_calc_ = 1.480 gcm^‑3^, F(000) = 664, µ = 2.657 mm^‑1^, T = 120(1) K, θ_max_ = 76.608°, 20958 total reflections, 2505 with I_o_ > 2σ(I_o_), R_int_ = 0.0590, 3003 data, 193 parameters, 0 restraints, GooF = 1.048, R = 0.0382 and wR = 0.0945 [I_o_ > 2σ(I_o_)], R = 0.0478 and wR =0.0990 (all reflections), 0.531<d∆ρ < -0.341eÅ^‑3^.

**Comments on Checkcif file**: There are no A- and B-alerts

Supplementary Fig. 39 Crystal structure of **(246TriMePyNOH)CH_3_SO_3_** (246TriMePyNO = 2,4,6-trimethylpyridine *N*-oxide) with the thermal displacement parameter at 50% probability level. Colour Key: yellow = sulphur, blue = nitrogen, red = oxygen, dark grey = carbon, and light cyan = hydrogen. Crystallization solvent = acetonitrile. Diffusion solvent = hexane.

**Crystal data for (246TriMePyNOH)CH_3_SO_3_:** CCDC-2529415, C_9_H_15_NO_4_S, M = 233.28 gmol^‑1^, colourless block, 0.13 × 0.12 × 0.1 mm^3^, triclinic, space group *P -1*, a = 8.1628(6) Å, b = 8.3224(5) Å, c = 9.2513(4) Å, α= 102.610(5) °, β = 92.832(5) °, γ = 111.577(6) °, V = 564.49(6) Å^3^, Z = 2, D_calc_ = 1.372 gcm^‑3^, F(000) = 248, µ = 2.543 mm^‑1^, T = 120(1) K, θ_max_ = 66.724 °, 6160 total reflections, 1800 with I_o_ > 2σ(I_o_), R_int_ = 0.0382, 1962 data, 141 parameters, 0 restraints, GooF = 1.108, R = 0.0387 and wR = 0.1093 [I_o_ > 2σ(I_o_)], R = 0.0414 and wR = 0.1114 (all reflections), 0.304 <d∆ρ < -0.434 eÅ^‑3^.

**Comments on Checkcif file**: There are no A- and B-alerts

Supplementary Fig. 40 Crystal structure of {**(246TriMePyNO)_2_H}PF_6_** (246TriMePyNO = 2,4,6-trimethylpyridine *N*-oxide) with the thermal displacement parameter at 50% probability level. Colour Key: light orange = phosphorous, light green = fluorine, blue = nitrogen, red = oxygen, dark grey = carbon, and light cyan = hydrogen. Crystallization solvent = dichloromethane. Diffusion = pentane.

**Crystal data for** {**(246TriMePyNO)_2_H}PF_6_:** CCDC-2529427, C_16_H_23_F_6_N_2_O_2_P, M = 420.33gmol^‑1^, colourless block, 0.176 × 0.114 × 0.077 mm^3^, monoclinic, space group *P2_1_/c*, a = 12.2717(2)Å, b = 24.1990(5)Å, c = 13.0078(2)Å, α= 90°, β = 103.410(2)°, γ = 90°, V = 3757.51(12)Å^3^, Z = 8, D_calc_ = 1.486 gcm^‑3^, F(000) = 1744, µ = 1.975 mm^‑1^, T = 120(1) K, θ_max_ = 66.747°, 36831 total reflections, 5347 with I_o_ > 2σ(I_o_), R_int_ = 0.0589, 6638 data, 499 parameters, 2 restraints, GooF = 1.042, R = 0.0413 and wR = 0.1085 [I_o_ > 2σ(I_o_)], R = 0.0530 and wR = 0.1158 (all reflections), 0.366<d∆ρ < -0.282eÅ^‑3^.

**Comments on Checkcif file**: There are no A- and B-alerts

Supplementary Fig. 41 Crystal structure of {**(3MeOPyNO)_2_H}PF_6_** (3MeOPyNO = 3-methoxypyridine *N*-oxide) with the thermal displacement parameter at 50% probability level. Colour Key: light orange = phosphorous, light green = fluorine, blue = nitrogen, red = oxygen, dark grey = carbon, and light cyan = hydrogen. Crystallization solvent = dichloromethane. Diffusion = hexane.

**Crystal data for** {**(3MeOPyNO)_2_H}PF_6_:** CCDC-2529451, C_12_H_15_F_6_N_2_O_2_P, M = 396.23 gmol^‑1^, colourless needle, 0.26 × 0.08 × 0.04 mm^3^, triclinic, space group *P-1*, a = 5.9383(7) Å, b = 7.5703(10) Å, c = 9.2124(9) Å, α= 78.988(10) °, β = 81.193(9) °, γ = 67.551(11) °, V = 374.24(8) Å^3^, Z = 1, D_calc_ = 1.758 gcm^‑3^, F(000) = 202, µ = 2.543 mm^‑1^, T = 120(1) K, θ_max_ = 66.745°, 3580 total reflections, 2724 with I_o_ > 2σ(I_o_), R_int_ = twin, 3580 data, 117 parameters, 0 restraints, GooF = 1.059, R = 0.0910 and wR = 0.2546 [I_o_ > 2σ(I_o_)], R = 0.1075 and wR = 0.2706 (all reflections), 0.981 <d∆ρ < -0.635 eÅ^‑3^.

**Comments on Checkcif file**: There are no A- and B-alerts

Supplementary Fig. 42 Crystal structure of {**(6)_2_H}PF_6_** with the thermal displacement parameter at 50% probability level. Colour Key: light orangee = phosphorous, light green = fluorine, blue = nitrogen, red = oxygen, dark grey = carbon, and light cyan = hydrogen. Crystallization solvent = dichloromethane. Diffusion = methyl *tert*-butyl ether.

**Crystal data for** {**(6)_2_H}PF_6_:** CCDC-2529447, C_12_H_15_F_6_N_2_O_4_P, M = 396.23 gmol^‑1^, colourless block, 0.11× 0.08 × 0.05 mm^3^, monoclinic, space group *I2/a*, a = 14.8355(3)Å, b = 6.7064(2)Å, c = 16.0306(4)Å, α= 90°, β = 94.416(2)°, γ = 90°, V = 1590.19(7)Å^3^, Z = 4, D_calc_ = 1.655gcm^‑3^, F(000) = 808, µ = 2.394 mm^‑1^, T = 120(1) K, θ_max_ = 66.735°, 9419 total reflections, 1279 with I_o_ > 2σ(I_o_), R_int_ = 0.0362, 1402 data, 125 parameters, 0 restraints, GooF = 1.104, R = 0.0668 and wR = 0.1803 [I_o_ > 2σ(I_o_)], R = 0.0707 and wR = 0.1837 (all reflections), 0.981<d∆ρ < -0.821eÅ^‑3^.

**Comments on Checkcif file**: There are no A- and B-alerts

Supplementary Fig. 43 Crystal structure of {**(2Me4MeOPyNO)_2_H}I_3_** (2Me4MeOPyNO = 2-methyl-4-methoxypyridine *N*-oxide) with the thermal displacement parameter at 50% probability level. Colour Key: purple = iodine, blue = nitrogen, red = oxygen, dark grey = carbon, and light cyan = hydrogen. Crystallization solvent = dichloromethane. Diffusion = pentane.

**Crystal data for** {**(2Me4MeOPyNO)_2_H}I_3_:** CCDC-2529441, C_14_H_19_I_3_N_2_O_4_, M = 660.01 gmol^‑1^, red plate, 0.16 × 0.04 × 0.03 mm^3^, triclinic, space group *P-1*, a = 7.9919(3) Å, b = 8.4008(3) Å, c = 9.2749(2) Å, α= 112.652(3) °, β = 100.273(2) °, γ = 110.144(3) °, V = 503.57(3) Å^3^, Z = 1, D_calc_ = 2.176 gcm^‑3^, F(000) = 308, µ = 36.725 mm^‑1^, T = 120(1) K, θ_max_ = 66.750°, 5411 total reflections, 1658 with I_o_ > 2σ(I_o_), R_int_ = 0.0296, 1781 data, 108 parameters, 0 restraints, GooF = 1.082, R = 0.0216 and wR = 0.0560 [I_o_ > 2σ(I_o_)], R = 0.0233 and wR = 0.0567 (all reflections), 0.595 <d∆ρ < -0.935 eÅ^‑3^.

**Comments on Checkcif file**: There are no A- and B-alerts

Supplementary Fig. 44 Crystal structure of **(2Me4MeOPyNO)_2_HPF_6_** (2Me4MeOPyNO = 2-methyl-4-methoxypyridine *N*-oxide) with the thermal displacement parameter at 50% probability level. Colour Key: light orange = phosphorus, light green = fluorine, red = oxygen, blue = nitrogen, dark grey = carbon, and light cyan = hydrogen. Crystallization solvent = dichloromethane. Diffusion solvent = pentane.

**Crystal data for (2Me4MeOPyNO)_2_HPF_6_:** CCDC-2529414, C_14_H_19_F_6_N_2_O_4_P, M = 424.28gmol^‑1^, colourless block, 0.32× 0.13× 0.07 mm^3^, monoclinic, space group *P2_1_/c*, a = 15.8634(2)Å, b = 14.9211(2)Å, c = 7.46430(10)Å, α= 90°, β = 96.8330(10)°, γ = 90°, V = 1754.25(4)Å^3^, Z = 4, D_calc_ = 1.606 gcm^‑3^, F(000) = 872, µ = 2.212mm^‑1^, T = 120(1) K, θ_max_ = 66.746°, 11719 total reflections, 2773 with I_o_ > 2σ(I_o_), R_int_ = 0.0306, 3080 data, 252 parameters, 2 restraints, GooF = 1.083, R = 0.0341 and wR = 0.0911 [I_o_ > 2σ(I_o_)], R = 0.0373and wR = 0.0934 (all reflections), 0.394<d∆ρ < -0.364eÅ^‑3^.

**Comments on Checkcif file**: There are no A- and B-alerts

Supplementary Fig. 45 Crystal structure of {**(3PhPyNO)_2_H}PF_6_** (3PhPyNO = 3-phenylpyridine *N*-oxide) with the thermal displacement parameter at 50% probability level. Colour Key: light orange = phosphorous, light green = fluorine, blue = nitrogen, red = oxygen, dark grey = carbon, and light cyan = hydrogen. Crystallization solvent = dichloroethane. Diffusion = pentane.

**Crystal data for** {**(3PhPyNO)_2_H}PF_6_:** CCDC-2529434, C_22_H_19_F_6_N_2_O_2_P, M = 488.36 gmol^‑1^, colourless block, 0.18× 0.09× 0.06 mm^3^, orthorhombic, space group *P2_1_2_1_2_1_*, a = 7.14030(10)Å, b = 11.1369(2)Å, c = 26.3450(5)Å, α= 90°, β = 90°, γ = 90°, V = 2094.98(6)Å^3^, Z = 4, D_calc_ = 1.548gcm^‑3^, F(000) = 1000, µ = 1.873 mm^‑1^, T = 120(1) K, θ_max_ = 66.746°, 16007 total reflections, 3359 with I_o_ > 2σ(I_o_), R_int_ = 0.0384, 3709 data, 302 parameters, 2 restraints, GooF = 1.124, R = 0.0301 and wR = 0.0706 [I_o_ > 2σ(I_o_)], R = 0.0379 and wR = 0.0841 (all reflections), 0.179<d∆ρ < -0.297eÅ^‑3^.

**Comments on Checkcif file**: There are no A- and B-alerts

Supplementary Fig. 46 Crystal structure of {**(4EtPyNO)_2_H}PF_6_** (4EtPyNO = 4-ethylpyridine *N*-oxide) with the thermal displacement parameter at 50% probability level. Colour Key: light orange = phosphorous, light green = fluorine, blue = nitrogen, red = oxygen, dark grey = carbon, and light cyan = hydrogen. Crystallization solvent = dichloromethane. Diffusion = diethyl ether.

**Crystal data for** {**(4EtPyNO)_2_H}PF_6_:** CCDC-2529429, C_14_H_19_F_6_N_2_O_2_P, M = 392.28 gmol^‑1^, colourless plate, 0.18× 0.1 × 0.06 mm^3^, monoclinic, space group *P2/n*, a = 19.6451(5) Å, b = 8.3747(2) Å, c = 21.3754(6) Å, α= 90°, β = 100.673(2) °, γ = 90°, V = 3455.88(16) Å^3^, Z = 8, D_calc_ = 1.508 gcm^‑3^, F(000) = 1616, µ = 2.105 mm^‑1^, T = 150(1) K, θ_max_ = 66.747 °, 40476 total reflections, 4921 with I_o_ > 2σ(I_o_), R_int_ = 0.0590, 6114 data, 479 parameters, 10 restraints, GooF = 1.034, R = 0.0661 and wR = 0.1820 [I_o_ > 2σ(I_o_)], R = 0.0791 and wR = 0.1936 (all reflections), 0.961 <d∆ρ < -0.335 eÅ^‑3^.

**Comments on Checkcif file**: There are no A- and B-alerts

Supplementary Fig. 47 Crystal structure of **(4)4CF_3_BzH** (4CF_3_BzH = 4-trifluoromethylbenzoic acid) with the thermal displacement parameter at 50% probability level. Colour Key: light green = fluorine, red = oxygen, blue = nitrogen, dark grey = carbon, and light cyan = hydrogen. Crystallization solvent = dichloroethane. Diffusion solvent = pentane.

**Crystal data for (4)4CF_3_BzH:** CCDC-2529421, C_14_H_12_F_3_NO_3_, M = 299.25 gmol^‑1^, colourless block, 0.16 × 0.11 × 0.08 mm^3^, triclinic, space group *P-1*, a = 10.7482(2) Å, b = 11.0046(2) Å, c = 11.9567(2) Å, α= 103.947(2)°, β = 98.566(2)°, γ = 100.130(2)°, V = 1323.77(4) Å^3^, Z = 4, D_calc_ = 1.501 gcm^‑3^, F(000) = 616, µ = 1.162 mm^‑1^, T = 120(1) K, θ_max_ = 66.748 °, 17528 total reflections, 4163 with I_o_ > 2σ(I_o_), R_int_ = 0.0287, 4683 data, 383 parameters, 0 restraints, GooF = 1.047, R = 0.0372 and wR = 0.0977 [I_o_ > 2σ(I_o_)], R = 0.0418 and wR = 0.1005 (all reflections), 0.253 <d∆ρ < -0.256 eÅ^‑3^.

**Comments on Checkcif file**: There are no A- and B-alerts

Supplementary Fig. 48 Crystal structure of **(7)DNBH** (DNBH = 3,5-dinitrobenzoic acid) with the thermal displacement parameter at 50% probability level. Colour Key: brown = bromine, red = oxygen, blue = nitrogen, dark grey = carbon, and light cyan = hydrogen. Crystallization solvent = dichloroethane. Diffusion solvent = pentane.

**Crystal data for (7)DNBH:** CCDC-2529454, C_12_H_8_BrN_3_O_7_, M = 386.12 gmol^‑1^, colourless block, 0.07 × 0.05 × 0.03 mm^3^, monoclinic, space group *P2_1_*, a = 3.82200(10) Å, b = 14.7066(4) Å, c = 12.3161(3) Å, α= 90 °, β = 95.822(3) °, γ = 90 °, V = 688.70(3) Å^3^, Z = 2, D_calc_ = 1.862 gcm^‑3^, F(000) = 384, µ = 4.510 mm^‑1^, T = 120(1) K, θ_max_ = 77.604 °, 4917 total reflections, 1899 with I_o_ > 2σ(I_o_), R_int_ = 0.0507, 2037 data, 209 parameters, 1 restraints, GooF = 1.066, R = 0.0353 and wR = 0.0847 [I_o_ > 2σ(I_o_)], R = 0.0389 and wR = 0.0863 (all reflections), 0.613 <d∆ρ < -0.429 eÅ^‑3^.

**Comments on Checkcif file**: There are no A-Alerts. There is a level B-alert. The level B-alert and our response are given below.

Alert level B

PLAT431_ALERT_2_B Short Inter HL..A Contact Br1	..O3	. 2.92 Ang.

1-x,-1/2+y,2-z =	2_647 Check

**Author Response: Halogen bonding**

PLAT915_ALERT_3_B No Flack x Check Done: Low Friedel Pair Coverage	38 %

**Author Response: There are enough Friedel pairs to obtain a reliable**

**Flack parameter due to the presence of a bromine atom.**

Supplementary Fig. 49 Crystal structure of {**(3IPyNO)_2_H}PF_6_** (3IPyNO = 3-iodopyridine *N*-oxide) with the thermal displacement parameter at 50% probability level. Colour Key: purple = iodine, light orange = phosphorous, light green = fluorine, red = oxygen, blue = nitrogen, dark grey = carbon, and light cyan = hydrogen. Crystallization solvent = dichloroethane, diffusion solvent = pentane.

**Crystal data for** {**(3IPyNO)_2_H}PF_6_:** CCDC-2529448, C_10_H_9_F_6_I_2_N_2_O_2_P, M = 587.96gmol^‑1^, colourless block, 0.23 × 0.21 × 0.08 mm^3^, triclinic, space group *P-1*, a = 6.1981(2)Å, b = 7.9410(3)Å, c = 8.3918(2)Å, α= 98.111(2)°, β = 108.053(2)°, γ = 95.306(3)°, V = 384.68(2)Å^3^, Z = 1, D_calc_ = 2.538 gcm^‑3^, F(000) = 274, µ = 33.827mm^‑1^, T = 120(1) K, θ_max_ = 66.723°, 7393 total reflections, 1346 with I_o_ > 2σ(I_o_), R_int_ = 0.0892, 1368 data, 107 parameters, 0 restraints, GooF = 1.085, R = 0.0416 and wR = 0.1064 [I_o_ > 2σ(I_o_)], R = 0.0419 and wR = 0.1071 (all reflections), 2.139<d∆ρ < -1.043 eÅ^‑3^.

**Comments on Checkcif file**: There are no A- and B-alerts

Supplementary Fig. 50 Crystal structure of **IsQNO-H-ClO_4_** (IsQNO = isoquinoline *N*-oxide) with the thermal displacement parameter at 50% probability level. Colour Key: green = chlorine, red = oxygen, blue = nitrogen, dark grey = carbon, and light cyan = hydrogen. Disordered parts were omitted for viewing clarity. Crystallization solvent = dichloromethane, diffusion solvent = pentane.

**Crystal data for IsQNO-H-ClO_4_:** CCDC-2529445, C_9_H_8_ClNO_5_, M = 245.61gmol^‑1^, colourless block, 0.27× 0.06× 0.04 mm^3^, triclinic, space group *P-1*, a = 6.6073(4)Å, b = 8.5697(6)Å, c = 9.8936(6)Å, α= 106.731(5)°, β = 103.423(6)°, γ = 103.423(6)°, V = 510.52(6)Å^3^, Z = 2, D_calc_ = 1.598gcm^‑3^, F(000) = 252, µ = 3.427 mm^‑1^, T = 120(1) K, θ_max_ = 66.724°, 5211 total reflections, 1571 with I_o_ > 2σ(I_o_), R_int_ = 0.0304, 1792 data, 176 parameters, 2 restraints, GooF = 1.254, R = 0.0835 and wR = 0.2545 [I_o_ > 2σ(I_o_)], R = 0.0895 and wR = 0.2685 (all reflections), 0.869<d∆ρ < -0.859eÅ^‑3^.

**Comments on Checkcif file**: There are no A- and B-alerts

Supplementary Fig. 51 Crystal structure of **4DMAPO** (**4DMAPO** = 4-dimethylaminopyridine *N*-oxide) with the thermal displacement parameter at 50% probability level. Colour Key: blue = nitrogen, red = oxygen, dark grey = carbon, and light cyan = hydrogen. Crystallization solvent = dichloroethane. Diffusion = hexane.

**Crystal data for 4DMAPO:** CCDC-2529422, C_7_H_14_N_2_O_3_, M = 174.20 gmol^‑1^, colourless block, 0.26 × 0.11 × 0.08 mm^3^, monoclinic, space group *P2_1_/c*, a = 8.3879(3) Å, b = 16.1280(4) Å, c = 6.7521(2) Å, α= 90°, β = 103.747(3)°, γ = 90°, V = 887.26(5) Å^3^, Z = 4, D_calc_ = 1.304 gcm^‑3^, F(000) = 376, µ = 0.855 mm^‑1^, T = 120(1) K, θ_max_ = 66.730 °, 11857 total reflections, 1441 with I_o_ > 2σ(I_o_), R_int_ = 0.0506, 1563 data, 117 parameters, 0 restraints, GooF = 1.054, R = 0.0411 and wR = 0.1183 [I_o_ > 2σ(I_o_)], R = 0.0434 and wR = 0.1204 (all reflections), 0.256 <d∆ρ < -0.312 eÅ^‑3^.

**Comments on Checkcif file**: There are no A- and B-alerts

**4.6 Pyridine *N*-oxide-diiodine halogen-bonded complexes**

Supplementary Fig. 52 Crystal structure of **1-I_2_** with the thermal displacement parameter at 50% probability level. Colour Key: purple = iodine, blue = nitrogen, red = oxygen, dark grey = carbon, and light cyan = hydrogen. Crystallization solvent = dichloroethane. Diffusion solvent = hexane.

**Crystal data for 1-I_2_:** CCDC-2529450, C_5_H_5_I_2_NO, M = 348.90 gmol^‑1^, red bloks, 0.08 × 0.06 × 0.04 mm^3^, triclinic, space group *P-1*, a = 9.0997(3) Å, b = 9.3927(4) Å, c = 10.6330(3) Å, α= 92.270(3) °, β = 90.134(2) °, γ = 106.881(3) °, V = 868.88(5) Å^3^, Z = 4, D_calc_ = 2.667 gcm^‑3^, F(000) = 624, µ = 56.316 mm^‑1^, T = 120(1) K, θ_max_ = 66.728 °, 10035 total reflections, 2663 with I_o_ > 2σ(I_o_), R_int_ = 0.0662, 3067 data, 163 parameters, 30 restraints, GooF = 1.063, R = 0.0439 and wR = 0.1148 [I_o_ > 2σ(I_o_)], R = 0.0504 and wR = 0.1235 (all reflections), 2.028 <d∆ρ < -2.136 eÅ^‑3^.

**Comments on Checkcif file**: There are no B-Alerts. There is a level A-alert. The level A-alert and our response are given below.

Alert level A

PLAT431_ALERT_2_A Short Inter HL..A Contact I3	..O2	. 2.69 Ang.

1-x,1-y,1-z =	2_666 Check

**Author Response: Halogen bonding**

Supplementary Fig. 53 Crystal structure of **3-I_2_** with the thermal displacement parameter at 50% probability level. Colour Key: purple = iodine, blue = nitrogen, red = oxygen, dark grey = carbon, and light cyan= hydrogen. Crystallization solvent = dichloromethane. Diffusion solvent = hexane.

**Crystal data for 3-I_2_:** CCDC-2529456, C_6_H_7_I_2_NO, M = 362.93 gmol^‑1^, red plate, 0.07× 0.052× 0.031mm^3^, triclinic, space group *P2_1_/c*, a = 8.1256(3) Å, b = 8.1277(3)Å, c = 8.5580(4)Å, α= 115.010(4)°, β = 105.028(4)°, γ = 98.810(3)°, V = 471.86(4)Å^3^, Z = 2, D_calc_ = 2.554 gcm^‑3^, F(000) = 328, µ = 51.889 mm^‑1^, T = 120(1) K, θ_max_ = 76.773°, 3747 total reflections, 3555 with I_o_ > 2σ(I_o_), R_int_ = twin, 3747data, 93 parameters, 0 restraints, GooF = 1.131, R = 0.0635and wR = 0.1917 [I_o_ > 2σ(I_o_)], R = 0.0647 and wR = 0.1941 (all reflections), 2.851<d∆ρ < -1.759eÅ^‑3^.

**Comments on Checkcif file**: There are no B-Alerts. There is a level A-alert. The level A-alert and our response are given below.

Alert level A

PLAT431_ALERT_2_A Short Inter HL..A Contact I2	..O1	. 2.68 Ang.

x,-1+y,z =	1_545 Check

**Author Response: Halogen bonding**

Supplementary Fig. 54 Partial packing structures of [O–I–O]⁺ complexes (a) **1-I** (b) **2-I** (c) **3-I** and (d) **4-I** displaying the *N*-oxide oxygen-based hydrogen bonding contacts. Packing structures of complexes **5-I** and **5-1a** are not attempted due to their disordered 2MeOPyNO.

Supplementary Fig. 55 Partial packing structures of [O–I–O]⁺ complexes (a) **6-I** and (b) **7-I** displaying the *N*-oxide oxygen-based hydrogen bonding and other contacts.

Supplementary Table 1. Reported [N–I–N]^+^ halogen-bonded complexes involving parent pyridines corresponding to the pyridine *N*-oxides examined in this study. As of 06 Feb 2026, a total of 101 [N–I–N]^+^ complexes are deposited in the Cambridge Structural Database (CSD). Only the structures listed in this table were selected for present study in order to elucidate the relative influence of pyridinic nitrogen versus *N*-oxide oxygen-based XBs.

| **Complex** | **CCDC No.** | **N−I (Å)** | **I−N (Å)** | **N···N (Å)** | **N−I−N (°)** |
| --- | --- | --- | --- | --- | --- |
| **{(Py)_2_I}PF_6_** | 1030916 | 2.253(8) | 2.253(8) | 4.51 | 180 |
| **{(2MePy)_2_I}PF_6_** | 2302774 | 2.262(7) | 2.262(7) | 4.52 | 180 |
| **{(3MePy)_2_I}PF_6_** | present study | 2.272(9) | 2.272(9) | 4.54 | 180 |
| **{(4MePy)_2_I}PF_6_** | 2064895 | 2.268(4) | 2.235(4) | 4.50 | 178.34(14) |
| **{(2MeOPy)_2_I}PF_6_** | present study | 2.293(3) | 2.265(3) | 4.56 | 176.36(8) |
| **{(4MeOPy)_2_I}PF_6_** | present study | 2.261(8) | 2.249(8) | 4.51 | 176.6(3) |
| **{(3BrPy)_2_I}PF_6_** | present study | 2.273(5) | 2.261(5) | 4.53 | 175.4(2) |

Supplementary Table 2. Reported [S–I–S]^+^ halogen-bonded complexes featuring sulfur-based halogen-bond acceptors. As of 06 Feb 2026, 14 [S–I–S]^+^ complexes are available in the Cambridge Structural Database (CSD). The structures summarized here were used for comparative analysis of sulfur-centered XB motifs.

| **CCDC No** | **S-I**  ***I-S-C(°)*** | **I-S**  ***I-S-C(°)*** | **S-I-S** | **S···S** |
| --- | --- | --- | --- | --- |
| 252518 | 2.597(4)  *105.2(5)* | 2.702(4)  *108.2(5)* | 171.32(12) | 5.28 |
| 253655 | 2.5961(15)  *100.4(2)* | 2.6596(14)  *104.14(19)* | 177.77(5) | 5.26 |
| 1171779 | 2.601(2)  103.3(3) | 2.634(2)  103.3(3) | 174.97(9) | 5.23 |
| 783327 | 2.623(5)  *103.5(6)* | 2.592(5)  *105.0(6)* | 175.40(18) | 5.21 |
| 1021797 | 2.651(2)  *104.4(2)* | 2.597(2)  *106.3(2)* | 174.74(5) | 5.24 |
| 1180503 | 2.6103(6)  *101.742(16)* | 2.6103(6)  *101.742(16)* | 180 | 5.21 |
| 1181004 | 2.6290(15)  *106.25(6)* | 2.6290(15)  *106.25(6)* | 180 | 5.26 |
| 2019054 | 2.6406(11)  *104.17(15)* | 2.5922(14)  *106.27(16)* | 177.03(4) | 5.23 |
| 150692 | 2.654(4)  *103.2(4)* | 2.602(4)  *104.3(5)* | 179.07(12) | 5.26 |
|  | 2.623(4)  *104.2(4)* | 2.634(4)  *103.1(4)* | 178.75(14) | 5.26 |
| 2008357 | 2.6300(15)  *100.1(2)* | 2.6300(15)  *100.0(2*) | 176.06(4) | 5.26 |
| 984540 | 2.6279(5)  *102.28(6)* | 2.6297(5)  *100.52(6)* | 178.119(15) | 5.26 |
| 727002 | 2.638(2)  *100.3(2)* | 2.6231(19)  *100.9(2)* | 178.93(6) | 5.26 |
| 813538 | 2.6445(14)  *99.60(19)* | 2.6242(14)  *100.28(19)* | 178.93(4) | 5.27 |
| 172151 | 2.654(6)  *102.7(7)* | 2.654(6)  *102.7(7)* | 180 | 5.31 |

Supplementary Table 3. Reported [O–I–O]^−^ halogen-bonded complexes featuring carboxylate oxygen atoms. As of 28 January 2026, 9 [O–I–O]^−^ complexes are available in the Cambridge Structural Database (CSD). Duplicates and disorder structures were not considered for analysis.

| **CCDC Number** | **O-I** | **I-O** | **O-I-O** | **O···O** |
| --- | --- | --- | --- | --- |
| 2105590** | 2.185(7) | 2.185(7) | 179.8(4) | 4.365(9) |
|  |  |  |  |  |
| 1038785 | 2.192(2) | 2.198(2) | 178.24(8) | 4.390(3) |
| 1960378 | 2.203(3) | 2.203(3) | 180 | 4.407(8) |
| 1960379 | 2.164(6) | 2.164(6) | 180 | 4.328(11) |
|  | 2.201(4) | 2.201(4) | 180 | 4.401(7) |
| 1496317 | 2.1835(18) | 2.1835(18) | 180 | 4.406(5) |
|  | 2.203(2) | 2.203(2) | 180 | 4.367(4) |
| 1496318 | 2.193(5) | 2.193(5) | 179.8(2) | 4.385(9) |
| 1496319* | --* | --* | --* | 4.375(16) |
| 1496320 | 2.1962(10) | 2.2021(11) | 176.62(4) | 4.3964(16) |

** alkoxide-based halogen bonded complex. *****disordered and incorrect structural solution, thus not considered in the present study.

**5. 1H,15N HMBC NMR Spectroscopy.**

**5.1 General information**

The CD_2_Cl_2_ purchased from Eurisotop was used as received and stored over 4 Å molecular sieves overnight prior to use. All NMR samples were prepared under an argon atmosphere using standard Schlenk-line techniques. NMR tubes were oven dried at 110 °C overnight before use. For ʹʹin-tubeʹʹ syntheses, 2 mg of the respective *N*‑oxide was employed, and iodine(I) complexes were prepared using a 2:1:1 molar ratio of *N*‑oxide, silver(I) salt, and elemental iodine. The elemental iodine used matched that in the crystallization procedures described above. All ^15^N NMR spectra were recorded on a Bruker Avance III 500 MHz spectrometer equipped with a Prodigy cryoprobe. An external reference containing 90% ^15^N enriched nitromethane sealed in a capillary was used. The nitromethane signal was set to 0 ppm, and the same acquisition parameters were applied to all samples except for the number of scans and the receiver gain. Under standard conditions (NS = 4 scans; TD = 2048/512; TD0 = 1; SW(ppm) = 9.99 and 599.79; D1 = 2 s), the total experiment time was ≈1 hour 19 minutes. However, for most complexes, ^15^N resonances were obtained within ~20 minutes, and experiments were halted at that point. In some cases, no ^15^N signal could be observed. For example, in Entry 7, no resonance was detected despite acquiring multiple datasets with relaxation delays of D1 = 4, 10, 20, and 30 s. Subsequently, Entry 8 was prepared using PF_6_ as the counter anion, and a ^15^N signal was observed. For Entry 5, 2.5 mg of crystals was added to 600 µL of CD_2_Cl_2_, but incomplete dissolution prevented signal acquisition under standard conditions (D1 = 2 s). A weak resonance was eventually detected when the relaxation delay was increased to D1 = 30 s.

The absolute ^15^N NMR coordination shifts are calculated as |Δδ^15^N_coord_| = Δδ^15^N_complex_ – Δδ^15^N_ligand_. Larger |Δδ^15^N_coord_| values indicative stronger halogen bonds. For PyNOs-silver(I) complexes, |Δδ^15^N_coord_| values range from 1 to 9 ppm . These values must be interpreted with caution because several PyNO-Ag(I) mixtures formed gels or precipitates in CD_2_Cl_2_, which compromises sample homogeneity and renders the corresponding ¹⁵N NMR data less reliable. Despite the limited number of iodine(I) complexes investigated, certain patterns in their ^15^N chemical shifts were observed. Parent PyNO and the methyl‑substituted derivatives all resonate between −112 and −119 ppm. In contrast, the methoxypyridine *N*‑oxides exhibit resonances in the −130 ppm region, consistent with the stronger electron‑donating nature of methoxy groups relative to methyl substituents. This increased electron donation leads to greater shielding of the nitrogen nucleus, thus shifting the ^15^N resonance upfield. In Entry 6 of the methoxy series, two distinct ^15^N resonances at −128 and −134 ppm were observed. Comparison with Entry 5 suggests that the signal at approximately −134 ppm corresponds to the iodine(I) complex, whereas the resonance at −128 ppm likely originates from a second iodine(I) complex featuring an alternative *N*-oxide conformation or coordination environment. More importantly, the −128 ppm signal is not attributable to protonated 2-methoxypyridine *N*-oxide, as the protonated ligand displays a characteristic ^15^N chemical shift at −150 ppm. Given the instability of these iodine(I) complexes, the present dataset does not allow for a comprehensive analysis of substituent, solvent, or concentration effects on halogen‑bond formation in solution. A more detailed solution NMR investigation, focusing on ligand electronics, steric effects, dynamic equilibria, and sample conditions, is planned for future work.

Supplementary Table 4. ^15^N NMR chemical shifts (δ in ppm) of PyNOs and their Δδ(^15^N) values (= δ^15^N_complex_ - δ^15^N_ligand_) silver(I), halogen(I), hydrogen-bonded and diiodine complexes in CD_2_Cl_2_

| **Entry** | **L** | **𝛿^15^N_L_** | 𝛿^15^N_Ag(I)_ | **\|Δδ^15^N_coord_\| _=_ 𝛿^15^N_Ag(I)_ - 𝛿^15^N_L_** | **𝛿^15^N_I(I)_** | **\|Δδ^15^N_coord_\| = 𝛿^15^N_I(I)_ - 𝛿^15^N_L_** | **𝛿^15^N_LH_** | **𝛿^15^N_LI2_** |
| --- | --- | --- | --- | --- | --- | --- | --- | --- |
| **1** | **1** | -84 | -88 | 4 | -112 | 28 | -129 | -88^a^ |
| **2** | **2** | -87 | -96.3 | 9.3 | -114.5 | 27.5 | -121 | -- |
| **3** | **3** | -85 | NS | - | -110,-119* | 25, 34 | -122 | -93^a^ |
| **4** | **4** | -90 | -96.4 | 6.4 | -117 | 27 | -126 | -- |
| **5** | **5** | -106 | NS | -- | -135 (-134.5)^b^ | 29 (28.5)^b^ | -150 | -- |
| **6** | **5** | -106 | NS | 1 | -128, -134 | 22, 28 |  | -- |
| **7** | **6** | -102 | -103 | 1 | NS | -- | -154 | -- |
| **8** | **6** | -102 | NS | -- | -131^c^ | 29 |  | -- |
| **9** | **7** | -79.5 | -81.2 | 1.7 | -109 | 29.5 | -120 | -- |
| **10** | **Pyridine** | -62 | -118 | 56 | -172 | 110 | -87 | -- |

Notes: NS = no signal. ^a^ 𝛿^15^N NMR of PyNO-diiodine crystals. ^b^ 𝛿^15^N NMR of iodine(I) complex crystals and this 𝛿^15^N value is same as the iodine(I) complexe when prepared in NMR tube. ^c^this is a 𝛿^15^N NMR of iodine(I) complex with PF_6_ anion.

**5.2 ^1^H, ^15^N HMBC NMR spectra**

Supplementary Fig. 56 ^1^H ^15^N HMBC NMR spectra for **1** in CD_2_Cl_2_.

Supplementary Fig. 57. ^1^H ^15^N HMBC NMR spectra for **2** in CD_2_Cl_2_.

Supplementary Fig. 58 ^1^H ^15^N HMBC NMR spectra for **3** in CD_2_Cl_2_.

Supplementary Fig. 59 ^1^H ^15^N HMBC NMR spectra for **4** in CD_2_Cl_2_.

Supplementary Fig. 60 ^1^H ^15^N HMBC NMR spectra for **5** in CD_2_Cl_2_.

Supplementary Fig. 61 ^1^H ^15^N HMBC NMR spectra for **6** in CD_2_Cl_2_.

Supplementary Fig. 62 ^1^H ^15^N HMBC NMR spectra for **7** in CD_2_Cl_2_.

Supplementary Fig. 63 ^1^H ^15^N HMBC NMR spectra for **pyridine** in CD_2_Cl_2_.

Supplementary Fig. 64 ^1^H ^15^N HMBC NMR spectra for **1-Ag** in CD_2_Cl_2_.

Supplementary Fig. 65 ^1^H ^15^N HMBC NMR spectra for **2-Ag** in CD_2_Cl_2_.

Supplementary Fig. 66 ^1^H ^15^N HMBC NMR spectra for **4-Ag** in CD_2_Cl_2_.

Supplementary Fig. 67 ^1^H ^15^N HMBC NMR spectra for **6-Ag** in CD_2_Cl_2_.

Supplementary Fig. 68 ^1^H ^15^N HMBC NMR spectra for **7-Ag** in CD_2_Cl_2_.

Supplementary Fig. 69 ^1^H ^15^N HMBC NMR spectra for {(**Py)_2_Ag}PF_6_** in CD_2_Cl_2_.

Supplementary Fig. 70 ^1^H ^15^N HMBC NMR spectra for **1-I** in CD_2_Cl_2_. Two ^15^N resonances are observed, one corresponding to the iodine(I) complex, and a second signal (marked with *) at −98 ppm, which is tentatively assigned to the **1-I_2_** halogen-bonded complex. This chemical shift is slightly downfield relative to the −88 ppm signal observed upon dissolution of crystalline **1-I_2_**. The difference may arise from additional hydrogen-bonding or secondary intermolecular interactions involving **1-I_2_** in solution.

Supplementary Fig. 71 ^1^H ^15^N HMBC NMR spectra for **2-I** in CD_2_Cl_2_.

Supplementary Fig. 72 The resonance marked with * is assigned to the iodine(I) complex. The intense signal at −120 ppm is attributed to a hydrogen-bonded species, as its chemical shift closely matches that of (**3H)PF_6_** (−122 ppm; see Table S4).

Supplementary Fig. 73 ^1^H ^15^N HMBC NMR spectra for **4-I** in CD_2_Cl_2_.

Supplementary Fig. 74 ^1^H ^15^N HMBC NMR spectra for **5-I** in tube reaction in CD_2_Cl_2_.

Supplementary Fig. 75 ^1^H ^15^N HMBC NMR spectra for **5-I** crystals dissolved in CD_2_Cl_2_.

Supplementary Fig. 76 ^1^H ^15^N HMBC NMR spectra for **5-Ia** in CD_2_Cl_2_. The peak marked with ‘*’ is likely a **5-I_2_** complex.

Supplementary Fig. 77 ^1^H ^15^N HMBC NMR spectra for **6-I** in CD_2_Cl_2_. The silver(I) complex ^1^H Signals marked with ‘*’ did not result in the ^15^N signal. However, the ^1^H signals marked with ‘*’ close match with silver(I) complex but not with protonated complex.

Supplementary Fig. 78 ^1^H ^15^N HMBC NMR spectra for **7-I** in CD_2_Cl_2_. Two ^15^N resonances are observed, one corresponding to the iodine(I) complex and a second signal (marked with *) at −91 ppm, tentatively assigned to the **7-I_2_** halogen-bonded complex. This assignment is supported by the similar chemical shifts observed for **1-I_2_** and **3-I_2_** in the same region (see Table S4).

Supplementary Fig. 79 ^1^H ^15^N HMBC NMR spectra for {**(pyridine)_2_I}PF_6_** in CD_2_Cl_2_.

Supplementary Fig. 80 ^1^H ^15^N HMBC NMR spectra for (**1H)PF_6_** in CD_2_Cl_2_

Supplementary Fig. 81 ^1^H ^15^N HMBC NMR spectra for {(**2)_2_H}PF_6_** in CD_2_Cl_2_. The ^15^N chemical shift observed upon dissolution of hydrogen-bonded {(**2)_2_H}PF_6_** crystals (Figure S20) is identical to that of hydrogen-bonded (**2H)PF_6_** crystals (Figure S21).

Supplementary Fig. 82 ^1^H ^15^N HMBC NMR spectra for (**2H)PF_6_** in CD_2_Cl_2_. The ^15^N chemical shift observed upon dissolution of hydrogen-bonded (**2H)PF_6_** crystals (Figure S21) matches that of hydrogen-bonded {(**2)_2_H}PF_6_** crystals (Figure S20).

Supplementary Fig. 83 ^1^H ^15^N HMBC NMR spectra for (**3H)PF_6_** in CD_2_Cl_2_.

Supplementary Fig. 84 ^1^H ^15^N HMBC NMR spectra for (**4H)PF_6_** in CD_2_Cl_2_. The spectra was acquired by dissolving hydrogen bonded crystals in CD_2_Cl_2_.

Supplementary Fig. 85 ^1^H ^15^N HMBC NMR spectra for (**5H)PF_6_** in CD_2_Cl_2_.

Supplementary Fig. 86 ^1^H ^15^N HMBC NMR spectra for (**6H)PF_6_** in CD_2_Cl_2_. The‘’doublet-like’’ ^15^N resonances at −147 and −154 ppm corresponds to protonated species. This behaviour could be due to solubility issues.

Supplementary Fig. 87 ^1^H ^15^N HMBC NMR spectra for (**7H)PF_6_** in CD_2_Cl_2_.

Supplementary Fig. 88 ^1^H ^15^N HMBC NMR spectra for (**PyH)PF_6_** in CD_2_Cl_2_.

**6. Density Functional Theory studies**

The DFT calculations were performed using the RI-PBE0^[8]^-D4^[9]^//def2-TZVP^[10,11]^ level of theory. For iodine, this basis set accounts for scalar relativistic effects using an effective core potential as described by Bergner and coworkers.^[12]^ To validate this approach, the zeroth-order regular approximation (ZORA) method^[13]^was employed for the [O-I-O]^+^ model. These validation calculations were conducted at the PBE0-D4/ZORA-def2-TZVP level using an all-electron basis set. Results obtained from the ZORA-PBE0-D4 basis set^[14]^ and Hamiltonian were consistent with the effective core potential data, as discussed in the main text.

The models for the [N-I-N]^+^, [O-I-O]^+^ and [S-I-S]^+^, were taken from the crystal structures as inputs and then fully optimized as a monomeric, positively charged unit using the TURBOMOLE 7.7^[15]^ software package. The resulting wavefunction was then subtracted and treated via the Multiwfn^[16]^ software to obtain the necessary cubes for plotting the MEP surfaces (using the Gaussview 6.0.16^[17]^ visualization software) and the AIM analysis (using the VMD^[18]^ software). The ELF plots were directly obtained by treating the wavefunction with the Multiwfn software, as well as the combined 2D-RDG plots. ETS-NOCV analysis and cubes were obtained utilizing the Multiwfn software and were plotted using the VMD visualization software. For the NBO analysis and NPA charges, the systems were treated at the RI-PBE0-D4//def2-TZVP level of theory using the NBO7.0^[19]^ extension of the orca 6.1.1^[20]^ software package and plotted using the VMD visualization program. The Mayer bond orders^[21]^ were obtained at the same level of theory. For the energy decomposition analysis (EDA), the calculations were performed at the RI-PBE0-D4/def2-TZVP level of theory using an improved Kitaura–Morokuma^[22]^ EDA scheme (DFT-EDA)^[23]^ as implemented in TURBOMOLE 7.7^[15]^ Each XB motif was partitioned into two fragments, [FRAGMENT 1, the I⁺ cation] and [FRAGMENT 2, the neutral N/O/S-donor unit], using the geometries of the corresponding [N–I–N]⁺/[O–I–O]⁺/[S–I–S]_1_⁺/[S–I–S]_2_⁺ complexes. The total interaction energy (E_int_) was decomposed into electrostatic (E_ele_), exchange-repulsion (Eexc-rep), orbital/polarization (E_orb_), correlation (E_cor_), and dispersion (E_disp_) contributions, as summarized in Table 3.

Supplementary Fig. 89 MEP surfaces of (a) [N–I–N]^+^ of pyridine, (b) [O–I–O]^+^ of pyridine N-oxide, (c) [S–I–S]^+^ of 2-mercapto-5-methyl-1,3,4-thiadiazole, and (d) [S–I–S]^+^ of H_2_C=S-I-S=CH_2_ halogen bonded complexes. The MEP surfaces are mapped onto the 0.001 e·bohr⁻³ electron density isosurface and are colour coded from red (more negative) to blue (more positive), with extremes given in kcal/mol. QTAIM analysis of halogen-bonded complexes (e) [N–I–N]^+^ of pyridine, (f) [O–I–O]^+^ of pyridine *N*-oxide, (g) [S–I–S]^+^ of 2-mercapto-5-methyl-1,3,4-thiadiazole, (h) [S–I–S]^+^ of 3-methylbenzothiazole-2-thione, and (i) [S–I–S]^+^ of H_2_C=S-I-S=CH_2_ showing bond critical points (BCPs) and corresponding –G/V ratios.

Supplementary Fig. 90 The optimized molecular geometry and corresponding 2D‑ELF map of [S–I–S]^+^ of 3-methylbenzothiazole-2-thione halogen‑bonded complex.

The 2D Reduced Density Gradient (RDG) analysis combined with the Laplacian of the electron density was employed to differentiate covalent, non-covalent, and intermediate (“transit”) interactions within the complexes. For the I^+^–N/O region, solid Laplacian isocontours appear between the interacting atoms, signifying a closed‑shell interaction. Notably, no characteristic RDG domains are detected within the applied density cutoff (ρ < 0.05 a.u.), indicating the absence of low-density non-covalent interaction signatures. The combination of a positive Laplacian and the lack of non-covalent interaction features suggests that the I^+^–N/O contact possesses a higher electron density than typical weak non‑covalent interactions, placing it firmly within an intermediate bonding regime, an assignment fully consistent with the corresponding –G/V values. A similar pattern is observed for the I^+^–S interaction. The presence of solid Laplacian isocontours again reflects a closed‑shell character, while the absence of low‑density RDG domains under the same cutoff confirms that this contact also exceeds the electron density usually associated with weak non-covalent interactions. This reinforces the classification of the I^+^–S interaction as an intermediate transit‑type interaction in agreement with the topological energy descriptors. In contrast, the ancillary S···I^+^ contacts exhibit distinct blue RDG isocontours between sulfur and iodine, clearly indicative of stabilizing non‑covalent interactions. These secondary contacts highlight the coexistence of both intermediate and weakly attractive interactions within the broader bonding environment of the system.

Supplementary Fig. 91 Two‑dimensional reduced density gradient (2D‑RDG) isosurfaces mapped onto the Laplacian of the electron density for the halogen‑bonded complexes, (a) [N–I–N]^+^ of pyridine, (b) [O–I–O]^+^ of pyridine *N*-oxide, (c) [S–I–S]^+^ of 2-mercapto-5-methyl-1,3,4-thiadiazole and [S–I–S]^+^ of 3-methylbenzothiazole-2-thione. The contour plots highlight regions of non-covalent interaction, with color scales indicating the sign(λ₂)ρ descriptor to distinguish attractive (blue-green) from repulsive (orange-red) interactions.

Supplementary Table 5. Mulliken, Löwdin, and Hirshfeld atomic charges for donor and acceptor atoms in the [N–I–N]^+^, [O–I–O]^+^, [S–I–S]^+^ (benzimidazole), and [S–I–S]^+^ (pyrrole) systems. The data highlight the differing charge distributions across N/O/S and iodine(I) within each halogen‑bonding framework.

| **XB framework** | **atom** | **Mulliken** | **Löewdin** | **Hirshfeld** |
| --- | --- | --- | --- | --- |
| [N–I–N]^+^ | N | -0.09139 | 0.090067 | -0.031969 |
|  | I | 0.135109 | 0.442782 | 0.109349 |
|  |  |  |  |  |
| [O–I–O]^+^ | O | -0.424471 | -0.166801 | -0.192897 |
|  | I | 0.198347 | 0.306145 | 0.063339 |
|  |  |  |  |  |
| [S–I–S]^+^ (benzimidazole) | S | -0.166251 | 0.333414 | -0.041282 |
|  | I | 0.078741 | 0.041459 | 0.025336 |
|  |  |  |  |  |
| [S–I–S]^+^ (pyrrole) | S | -0.129741 | 0.335666 | -0.043116 |
|  | I | 0.068024 | 0.035048 | 0.023966 |

**6.1 Cartesian coordinates for the [N-I-N]^+^ optimized monomer**

I	-1.5367858	-0.0556793	0.0001879

N	-3.7137021	-0.0556793	0.5772096

C	-4.6472852	-0.0556793	-0.3759825

C	-5.9911738	-0.0556793	-0.0636941

C	-6.3681842	-0.0556793	1.2701134

C	-5.3874389	-0.0556793	2.2495564

C	-4.0621337	-0.0556793	1.8652638

H	-4.2948413	-0.0556793	-1.4001747

H	-6.7244421	-0.0556793	-0.8593867

H	-7.4162361	-0.0556793	1.5435764

H	-5.6389423	-0.0556793	3.3019465

H	-3.2543302	-0.0556793	2.5868436

N	0.6401100	-0.0556793	-0.5767708

C	1.5737162	-0.0556793	0.3764501

C	2.9176097	-0.0556793	0.0642602

C	3.2947071	-0.0556793	-1.2695026

C	2.3140429	-0.0556793	-2.2490015

C	0.9887315	-0.0556793	-1.8648101

H	1.2211816	-0.0556793	1.4006139

H	3.6507696	-0.0556793	0.8599211

H	4.3428160	-0.0556793	-1.5429011

H	2.5656248	-0.0556793	-3.3012825

H	0.1809723	-0.0556793	-2.5864361

**6.2 Cartesian coordinates for the [O-I-O]^+^ optimized monomer**

I	-1.1617096	-0.4073602	-0.0596566

O	-1.6992254	-0.0626838	-2.1636597

O	-0.6169392	-0.7182874	2.0469000

N	-0.8059451	-0.5045009	-3.0368033

N	-1.6189930	-1.1116735	2.8193847

C	0.3188077	0.1980960	-3.2486938

H	0.4267486	1.0868813	-2.6435347

C	1.2267940	-0.2270373	-4.1904002

H	2.1254318	0.3536652	-4.3495391

C	0.9695266	-1.3793085	-4.9174615

H	1.6721488	-1.7240451	-5.6653593

C	-0.2012200	-2.0813631	-4.6764974

H	-0.4438630	-2.9823941	-5.2237756

C	-1.0801764	-1.6263993	-3.7207656

H	-2.0172711	-2.0988600	-3.4629929

C	-1.6834669	-2.4037536	3.1771747

H	-0.9078087	-3.0303494	2.7608542

C	-2.6776831	-2.8338015	4.0254671

H	-2.7080971	-3.8771671	4.3094414

C	-3.6133207	-1.9258787	4.4968123

H	-4.4020260	-2.2477248	5.1648896

C	-3.5230823	-0.5990095	4.1043186

H	-4.2292238	0.1430584	4.4519103

C	-2.5099481	-0.2085007	3.2596650

H	-2.3521211	0.8015692	2.9093218

**6.3 Cartesian coordinates for the [S-I-S]^+^ (2-mercapto-5-methyl-1,3,4-thiadiazole) optimized monomer**

I	-3.9107187	2.8173414	8.6524117

S	-1.4315036	0.8673629	7.0737988

S	-4.4388720	0.5030252	7.5599053

S	-5.6052381	5.7742737	7.7422471

S	-3.3840072	5.1251934	9.7564420

N	-2.7020409	-1.1351052	6.3771142

H	-3.4255884	-1.8200460	6.2067580

N	-1.4579896	-1.4257735	5.9595165

N	-4.5676508	7.4532247	9.2301852

H	-4.0034717	7.9255009	9.9231344

N	-5.4636302	8.1885365	8.5499274

C	-2.9260726	0.0271694	6.9931050

C	-0.6550235	-0.4643453	6.2513305

C	0.7891284	-0.4693986	5.9296265

H	1.0335786	-1.4035499	5.4262980

H	1.3904675	-0.3846203	6.8370213

H	1.0452102	0.3662644	5.2750553

C	-4.4652926	6.1486960	8.9695833

C	-6.1035856	7.4488299	7.7146939

C	-7.1627036	7.9501801	6.8114085

H	-7.2893537	9.0188917	6.9773418

H	-8.1108219	7.4440194	7.0049495

H	-6.8966507	7.7798074	5.7661451

**6.4 Cartesian coordinates for the [S-I-S]^+^ (3-methylbenzothiazole-2-thione) optimized monomer**

I	0.8769851	0.4402975	-0.0421620

S	-1.4585514	1.2966719	0.7515235

S	-1.8643267	0.5678494	-2.1537161

S	3.2301798	-0.4408609	-0.7532588

S	2.1257330	0.8837811	-3.2343031

N	-3.7566527	1.2769559	-0.6145874

N	4.4029468	-0.2383137	-3.1486020

C	-2.4323716	1.0694308	-0.6085787

C	-4.5082746	1.6917397	0.5536807

H	-3.8942490	1.5523463	1.4402681

H	-5.4040047	1.0774933	0.6382228

H	-4.7880996	2.7430356	0.4682301

C	-4.3692531	1.0715572	-1.8446325

C	-5.7150540	1.2306373	-2.1434280

H	-6.4250293	1.5579658	-1.3952187

C	-6.1242808	0.9629959	-3.4372934

H	-7.1694054	1.0788160	-3.6951749

C	-5.2184145	0.5491948	-4.4123610

H	-5.5684176	0.3463064	-5.4165451

C	-3.8761705	0.3956836	-4.1156020

H	-3.1710112	0.0753149	-4.8721600

C	-3.4625637	0.6642378	-2.8202979

C	3.3420112	0.0233759	-2.3722000

C	5.5839499	-0.9451927	-2.6930907

H	5.4359280	-1.2649347	-1.6648675

H	6.4506186	-0.2847225	-2.7446070

H	5.7495839	-1.8204241	-3.3222060

C	4.2970234	0.2317043	-4.4513220

C	5.2406437	0.1059797	-5.4604845

H	6.1844325	-0.3958048	-5.2912220

C	4.9377609	0.6473824	-6.6967033

H	5.6577003	0.5631493	-7.5010356

C	3.7272067	1.2993118	-6.9243991

H	3.5189830	1.7146622	-7.9022982

C	2.7877248	1.4261425	-5.9171072

H	1.8487719	1.9363000	-6.0912644

C	3.0875534	0.8835043	-4.6777234

**7. References**

[1]	D. B. G. Williams, M. Lawton, “Drying of organic solvents: Quantitative evaluation of the efficiency of several desiccants” *Journal of Organic Chemistry* **2010**, *75*, 8351–8354.

[2]	V. H. L. Wong, S. V. C. Vummaleti, L. Cavallo, A. J. P. White, S. P. Nolan, K. K. M. Hii, “Synthesis, Structure and Catalytic Activity of NHC–AgICarboxylate Complexes” *Chemistry - A European Journal* **2016**, *22*, 13320–13327.

[3]	M. J. Tanga, J. E. Bupp, T. K. Tochimoto, “Syntheses of five potential heterocyclic amine food mutagens” *J. Heterocycl. Chem.* **1997**, *34*, 717–727.

[4]	G. M. Sheldrick, “SHELXT - Integrated space-group and crystal-structure determination” *Acta Crystallogr. A* **2015**, *71*, 3–8.

[5]	O. V. Dolomanov, L. J. Bourhis, R. J. Gildea, J. A. K. Howard, H. Puschmann, “OLEX2: A complete structure solution, refinement and analysis program” *J. Appl. Crystallogr.* **2009**, *42*, 339–341.

[6]	G. M. Sheldrick, “A short history of SHELX” *Acta Crystallogr. A* **2008**, *64*, 112–122.

[7]	G. M. Sheldrick, “Crystal structure refinement with SHELXL” *Acta Crystallogr. C Struct. Chem.* **2015**, *71*, 3–8.

[8]	C. Adamo, V. Barone, “Toward reliable density functional methods without adjustable parameters: The PBE0 model” *Journal of Chemical Physics* **1999**, *110*, 6158–6170.

[9]	E. Caldeweyher, S. Ehlert, A. Hansen, H. Neugebauer, S. Spicher, C. Bannwarth, S. Grimme, “A generally applicable atomic-charge dependent London dispersion correction” *Journal of Chemical Physics* **2019**, *150*, 154122.

[10]	F. Weigend, R. Ahlrichs, “Balanced basis sets of split valence, triple zeta valence and quadruple zeta valence quality for H to Rn: Design and assessment of accuracy” *Physical Chemistry Chemical Physics* **2005**, 3297–3305.

[11]	F. Weigend, “Accurate Coulomb-fitting basis sets for H to Rn” *Physical Chemistry Chemical Physics* **2006**, *8*, 1057–1065.

[12]	S. G. Balasubramani, G. P. Chen, S. Coriani, M. Diedenhofen, M. S. Frank, Y. J. Franzke, F. Furche, R. Grotjahn, M. E. Harding, C. Hättig, A. Hellweg, B. Helmich-Paris, C. Holzer, U. Huniar, M. Kaupp, A. Marefat Khah, S. Karbalaei Khani, T. Müller, F. Mack, B. D. Nguyen, S. M. Parker, E. Perlt, D. Rappoport, K. Reiter, S. Roy, M. Rückert, G. Schmitz, M. Sierka, E. Tapavicza, D. P. Tew, C. Van Wüllen, V. K. Voora, F. Weigend, A. Wodyński, J. M. Yu, “TURBOMOLE: Modular program suite for ab initio quantum-chemical and condensed-matter simulations” *Journal of Chemical Physics* **2020**, *152*, 184107.

[13]	A. Bergner, M. Dolg, W. Küchle, H. Stoll, H. Preuß, “Ab initio energy-adjusted pseudopotentials for elements of groups 13-17” *Mol. Phys.* **1993**, *80*, 1431–1441.

[14]	E. Van Lenthe, E. J. Baerends, J. G. Snijders, “Relativistic regular two-component Hamiltonians” *J. Chem. Phys.* **1993**, *99*, 4597–4610.

[15]	D. A. Pantazis, X. Y. Chen, C. R. Landis, F. Neese, “All-electron scalar relativistic basis sets for third-row transition metal atoms” *J. Chem. Theory Comput.* **2008**, *4*, 908–919.

[16]	T. Lu, F. Chen, “Multiwfn: A multifunctional wavefunction analyzer” *J. Comput. Chem.* **2012**, *33*, 580–592.

[17],	R. Dennington, T. Keith, J. Millam, 2019, Semichem Inc preprint.

[18]	W. Humphrey, A. Dalke, K. Schulten, *VMD: Visual Molecular Dynamics*, **1996**.

[19],	E. D. Glendening, J. K. Badenhoop, A. E. Reed, J. E. Carpenter, J. A. Bohmann, C. M. Morales, P. Karafiloglou, C. R. Landis, F. Weinhold, 2018, Theoretical Chemistry Institute, University of Wisconsin preprint.

[20]	F. Neese, “The ORCA program system” *Wiley Interdiscip. Rev. Comput. Mol. Sci.* **2012**, *2*, 73–78.

[21]	I. Mayer, “Bond order and valence indices: A personal account” *J. Comput. Chem.* **2007**, *28*, 204–221.

[22]	P. Su; H. Li, Energy decomposition analysis of covalent bonds and intermolecular interactions *J. Chem. Phys*. **2009**, 131, 014102.

[23]	K. Kitaura, K. Morokuma, A new energy decomposition scheme for molecular interactions within the Hartree-Fock approximation, *Int. J. Quantum Chem*. **1976**, *10*, 325-340.
